# A comparative study of the gut microbiota in immune-mediated inflammatory diseases—does a common dysbiosis exist?

**DOI:** 10.1186/s40168-018-0603-4

**Published:** 2018-12-13

**Authors:** Jessica D. Forbes, Chih-yu Chen, Natalie C. Knox, Ruth-Ann Marrie, Hani El-Gabalawy, Teresa de Kievit, Michelle Alfa, Charles N. Bernstein, Gary Van Domselaar

**Affiliations:** 1 Department of Internal Medicine, University of Manitoba, Winnipeg, MB Canada; 2 University of Manitoba IBD Clinical and Research Centre, Winnipeg, MB Canada; 3 National Microbiology Laboratory, Public Health Agency of Canada, 1015 Arlington Street, Winnipeg, MB R3E 3R2 Canada; 4 Department of Medical Microbiology and Infectious Diseases, University of Manitoba, Winnipeg, MB Canada; 5 Department of Community Health Sciences, University of Manitoba, Winnipeg, MB Canada; 6 Arthritis Centre, University of Manitoba, Winnipeg, MB Canada; 7 Department of Microbiology, University of Manitoba, Winnipeg, MB Canada; 8 Department of Laboratory Medicine and Pathobiology, University of Toronto, Toronto, Canada

**Keywords:** Gut microbiota, Inflammatory bowel disease, Rheumatoid arthritis, Multiple sclerosis, 16S rRNA gene amplicon sequencing, Immune-mediated inflammatory disease, Bacteria, Machine learning classifiers, Taxonomic biomarkers

## Abstract

**Background:**

Immune-mediated inflammatory disease (IMID) represents a substantial health concern. It is widely recognized that IMID patients are at a higher risk for developing secondary inflammation-related conditions. While an ambiguous etiology is common to all IMIDs, in recent years, considerable knowledge has emerged regarding the plausible role of the gut microbiome in IMIDs. This study used 16S rRNA gene amplicon sequencing to compare the gut microbiota of patients with Crohn’s disease (CD; *N* = 20), ulcerative colitis (UC; *N* = 19), multiple sclerosis (MS; N = 19), and rheumatoid arthritis (RA; *N* = 21) versus healthy controls (HC; *N* = 23). Biological replicates were collected from participants within a 2-month interval. This study aimed to identify common (or unique) taxonomic biomarkers of IMIDs using both differential abundance testing and a machine learning approach.

**Results:**

Significant microbial community differences between cohorts were observed (pseudo *F* = 4.56; *p* = 0.01). Richness and diversity were significantly different between cohorts (pFDR < 0.001) and were lowest in CD while highest in HC. Abundances of *Actinomyces*, *Eggerthella, Clostridium III*, *Faecalicoccus*, and *Streptococcus* (pFDR < 0.001) were significantly higher in all disease cohorts relative to HC, whereas significantly lower abundances were observed for *Gemmiger*, *Lachnospira*, and *Sporobacter* (pFDR < 0.001). Several taxa were found to be differentially abundant in IMIDs versus HC including significantly higher abundances of *Intestinibacter* in CD, *Bifidobacterium* in UC, and unclassified *Erysipelotrichaceae* in MS and significantly lower abundances of *Coprococcus* in CD, *Dialister* in MS, and *Roseburia* in RA. A machine learning approach to classify disease versus HC was highest for CD (AUC = 0.93 and AUC = 0.95 for OTU and genus features, respectively) followed by MS, RA, and UC. *Gemmiger* and *Faecalicoccus* were identified as important features for classification of subjects to CD and HC. In general, features identified by differential abundance testing were consistent with machine learning feature importance.

**Conclusions:**

This study identified several gut microbial taxa with differential abundance patterns common to IMIDs. We also found differentially abundant taxa between IMIDs. These taxa may serve as biomarkers for the detection and diagnosis of IMIDs and suggest there may be a common component to IMID etiology.

**Electronic supplementary material:**

The online version of this article (10.1186/s40168-018-0603-4) contains supplementary material, which is available to authorized users.

## Background

Immune-mediated inflammatory diseases (IMIDs) are clinically heterogeneous diseases that share common pathogenic mechanisms and suspected etiologies. More than one hundred different diseases are described as immune-mediated and inflammatory; notable examples include inflammatory bowel disease (IBD), multiple sclerosis (MS), rheumatoid arthritis (RA), psoriasis, ankylosing spondylitis, and systemic lupus erythematosus. Globally, IMIDs affect 2 – 5% of the population; in Western populations, the prevalence of IMIDs ranges from approximately 5 – 8% [1]. The global incidence of many IMIDs is on the rise [2], though the reason for this increase is unclear.

Despite extensive investigations of the possible mechanisms of IMID pathogenesis, their etiologies are not clearly defined, though host genetic susceptibilities and environmental risk factors are thought to be involved. Relatively low disease concordance rates are observed in monozygotic twins [3]; hence, it is thought that environmental determinants have a substantial role in IMID etiopathogenesis. Environmental factors such as hygiene, socioeconomic status, vitamin D exposure, cigarette smoking, use of antibiotics (and other medications), and microbial exposure have been found to correlate with IMIDs [4–6]; however, no clear causal associations have been demonstrated to date. Several reports [7–10] suggest that individuals with a specific IMID are more susceptible to acquiring a secondary IMID, suggesting that IMIDs may share common etiological components.

Over the past decade, our understanding of IMIDs has been transformed by a growing appreciation of the pivotal role of the human microbiome. The microbiome composition has been reported to correlate with IMIDs such as IBD [11, 12], MS [13, 14], and RA [15, 16], both in human studies and in animal models of these diseases. Gut microbiota dysbiosis is commonly observed in IMIDs; however, its role in disease pathogenesis is still unknown. The influence of particular characteristics of gut microbiota dysbiosis have been widely reported for some IMID such as IBD; however, evidence has begun to unfold for the role of some taxa in IMIDs not considered to be gastrointestinal diseases, such as MS and RA.

We performed a pilot study to investigate the association of the gut microbiota with IMIDs including Crohn’s disease (CD), ulcerative colitis (UC), MS, and RA. To characterize the gut microbiota, we used 16S ribosomal RNA (rRNA) gene amplicon sequencing to assess the abundance of taxonomic groups in IMID and healthy controls (HC). Differential abundance testing and a supervised machine learning approach using a random forest algorithm were conducted and results compared to identify taxonomic biomarkers common among or specific to IMIDs.

## Methods

### Patients and stool sample collection

The study cohort is described in Table 1. For our disease cohorts, we chose patients with CD, UC, MS, and RA, since these diseases are all T cell-mediated yet preferentially target distinct organs. Moreover, previous studies have reported significantly increased odds of developing MS or RA in the presence of IBD [10] suggestive of a common etiological component. Research and ethics approval was obtained from the University of Manitoba’s Research Ethics Board. Patients with an IMID were recruited between 2010 and 2012 from the IBD Clinical and Research Centre and the Rheumatology ambulatory care clinic, both located at the Health Sciences Centre, Winnipeg, Canada*.* IMID patients were included if they met the standard criteria for case definition, i.e., Montreal Classification for IBD [17], 2010 McDonald criteria for MS [18], and 2010 American College of Rheumatology classification criteria for RA [19]; were over 18 years of age; and had not taken antibiotics in the previous 8 weeks. HC were recruited at the University of Manitoba Health Sciences Centre. For our HC cohort, we enrolled adults who had not taken antibiotics in the previous 8 weeks and had no medical history of gastrointestinal, neurological, or joint disease. Each participant self-collected two stool specimens approximately 2 months apart. The stool samples were kept refrigerated at 4 °C until transport. The stool was transported to the laboratory on ice and stored at − 80 °C until processing.

**Table 1:** Patient data at time of sample procurement

Disease	Average age, years^a^	*N* (female/male)^a^
Crohn’s disease	49.9	20 (14/5)
Ulcerative colitis	51.2	19 (11/8)
Multiple sclerosis	47.3	19 (14/4)
Rheumatoid arthritis	62.3	21 (14/7)
Healthy controls	32.4	23 (12/11)

^a^Tabulated metadata does not include information from patients whose metadata was not available

### DNA extraction, library preparation, and 16S rRNA gene amplicon sequencing

Stool specimens were thawed at 4 °C and were diluted 1:3 with milliQ water to create a stool slurry. DNA was isolated using the ZR-96 Fecal DNA Kit (Zymo Research, Irvine, CA) following a validated protocol [20]. The Illumina MiSeq sequencing library preparation protocol for 16S rRNA gene amplicons was followed with modifications [20]. Briefly, the 16S rRNA V4 region was amplified using primers 515fXT (GTGBCAGCMGCCGCGGTAA) and 806rXT (GGACTACHVGGGTWTCTAAT). Quality control, quantification, normalization, pooling, and sequencing of the library was performed as previously described [20]. Approximately 11 pM of the pooled, multiplexed samples were mixed with 37.5% PhiX spike-in control DNA and sequenced on an Illumina MiSeq instrument to generate 2 × 300 bp reads. Four MiSeq runs were performed with 56 multiplexed samples per run. In total, 224 samples were sequenced including technical replicates, mock communities of known composition (HM-782D; BEI Resources, Manassas, VA) and no-template controls.

### Data analysis

The mothur software suite (v. 1.39.5) was used to analyze 16S rRNA gene amplicon data. The specific analytical method is available as a Jupyter notebook (https://github.com/phac-nml/imid_microbiome). Paired-end reads were assembled into contigs using Needleman-Wunsch pairwise alignments. Only contigs containing both the forward and reverse V4 amplicon primer sequences with two or less nucleotide differences were retained. Primer sequences were trimmed from the resulting contigs. Contigs were discarded if they exceeded 275 bp in length, contained homopolymers exceeding 8 nucleotides in length, or contained ambiguous bases. A custom reference alignment specific to the 16S rRNA V4 region was created by trimming the curated 16S rDNA SILVA reference alignment (v. 128) to the region of interest [21]. Contigs were aligned to the aforementioned reference database. Briefly, the kmer search method (*k* = 8) was applied to identify the best (de-gapped) reference database sequence match followed by a pairwise alignment using the Needleman-Wunsch pairwise alignment method. Gaps not inserted in the pairwise alignment step were re-inserted into the query sequence using the NAST algorithm to mirror the aligned (gapped) reference database. Contigs aligning outside of the 16S rRNA amplified V4 region were removed. Aligned contigs that differed by a maximum of 2 bp were merged together to reduce the number of erroneous operational taxonomic units (OTUs) generated due to sequencing errors. In addition, chimeric artifacts (identified by UCHIME) [22] were also removed.

Taxonomic classification of sequences was performed using the naïve Bayesian classification method [23] and training set (v. 16) from RDP [24]. A minimum threshold bootstrap value of 60% was applied. Sequences identified as likely contaminants such as chloroplast and mitochondria or unwanted lineages such as archaea, eukaryota, or unknown (at the domain level) were removed. Sequences were clustered into OTUs based on a sequence similarity (≥ 97%) using the average neighbor algorithm and consensus taxonomy for each OTU was determined.

### Evaluation of Gram-negative abundance

Gram-negative microorganisms (i.e., Bacteroidetes, Proteobacteria, and Verrucomicrobia) were detected in a lower than expected abundance (discussed below). Accordingly, for differential abundance testing and machine learning classifications, the use of Gram-positive-only data was compared to the full (i.e., Gram-positive and Gram-negative) dataset.

### Processing and statistical analysis of operational taxonomic units

Following quality control and filtering, a total of 7943 OTUs, corresponding to 426 genera, was obtained. For analyses based on all phyla, the OTU count table was filtered to keep only taxa with non-zero OTU counts in at least 40 samples (the minimum number of samples for each cohort). This resulted in 426 OTUs corresponding to 118 genera. For analyses based on Gram-positive phyla, only OTUs within the phyla Firmicutes, Actinobacteria, and Tenericutes were used, and the same filtering criteria resulted in 383 OTUs and 90 genera. The OTU counts were then normalized at the 75^th^ percentile using the cumulative-sum scaling approach from the R “metagenomeSeq” [25] package. We refer to the normalized OTU counts as “abundance.” Genus-level taxonomic classifications of OTUs were extracted and abundances summarized. Genus abundance was calculated as the sum of OTU abundances belonging to the same genus. Downstream analyses included the analysis of both genus and OTU abundances. This approach was chosen to avoid the compositional nature of “relative abundance” (sum to one per sample) and the potential bias that can be created by highly abundant taxa [26]. A Kruskal-Wallis test and post hoc Dunn’s test with false discovery rate (FDR) correction for multiple comparisons were performed to compare median similarities of genus data among cohorts. Adjusted *p* values were considered significant at *p* < 0.05. Genus abundances were also analyzed using LEfSe [27] with an LDA score > 3 and a one-against-all multi-class analysis.

### Machine learning classification

Random forest machine learning classification was conducted using the “randomForest” R package [28] with 500 trees and the number of randomly sampled variables as the square root of the variable counts. Classifiers were built based on samples from the first time point and excluded technical replicates to avoid inflation of performance evaluation. We evaluated both OTU and genus OTU abundances as training data features to assess the performance at different levels of taxonomic specificity. In addition, we also evaluated all detected phyla versus Gram-positive phyla only to assess the relative ability of Gram-positive phyla data to classify the cohorts. When classifying diseased samples versus HC, a stratified sampling approach was used with the minimum number of samples of the classes (23 from HC). This technique, known as under-sampling [29], is employed when building decision trees with unbalanced sample counts. As each tree in the classifier is fit from a bootstrap sample set from the training data, samples that were not used to fit the corresponding tree were used to calculate the out-of-bag (OOB) error to assess model performance. For each pairwise comparison, the average OOB performance from ten models with different seeds was reported as balanced accuracy (BA) and area under the receiver operating characteristic curve (AUC). Feature importance averaged from the ten runs was reported as a measure of the mean decrease in Gini index (the impurity function) and visualized using the “ComplexHeatmap” R package [30]. All OTU post-processing (filtering and normalization) and machine learning analyses were conducted using R (v. 3.3.2, 3.3.3; R Development Core Team) and Bioconductor 3.4 [31].

### Diversity

Alpha diversity estimates including richness (ACE, Chao1) and diversity (Shannon, Simpson) were calculated using raw count data in mothur and visualized in phyloseq [32]. Alpha diversity measures were compared between disease cohorts using non-parametric Kruskal-Wallis tests and post hoc Dunn’s test with FDR correction for multiple comparisons. Microbiota community structures were visualized using principal coordinate analysis (PCoA) and tested using permutational analysis of variance (PERMANOVA) [33] implemented using the adonis function from the R “vegan” [34] package with the Bray-Curtis method [35] to calculate pairwise distances and 999 permutations.

## Results

The average base-calling error rate assessed by the co-sequenced mock communities was 0.01%, equivalent to six errors per 2 × 300 bp paired-end reads. A total of 31,315,771 raw sequences were assembled. Filtering out low quality, chimeric, and non-bacterial sequences generated 26,494,139 high-quality contigs. Sequences were clustered into 7943 OTUs based on their shared sequence similarity at a 97% threshold. The average number of sequences per sample was 120,621 ± 37,472 (range 20,165–296,780). Following the removal of Gram-negative phyla and normalization, the average number of sequences per sample was 86,865 ± 61,806 (range 14,671–438,203).

### Community structure, richness, and diversity of the gut microbiota

**Fig. 1:**
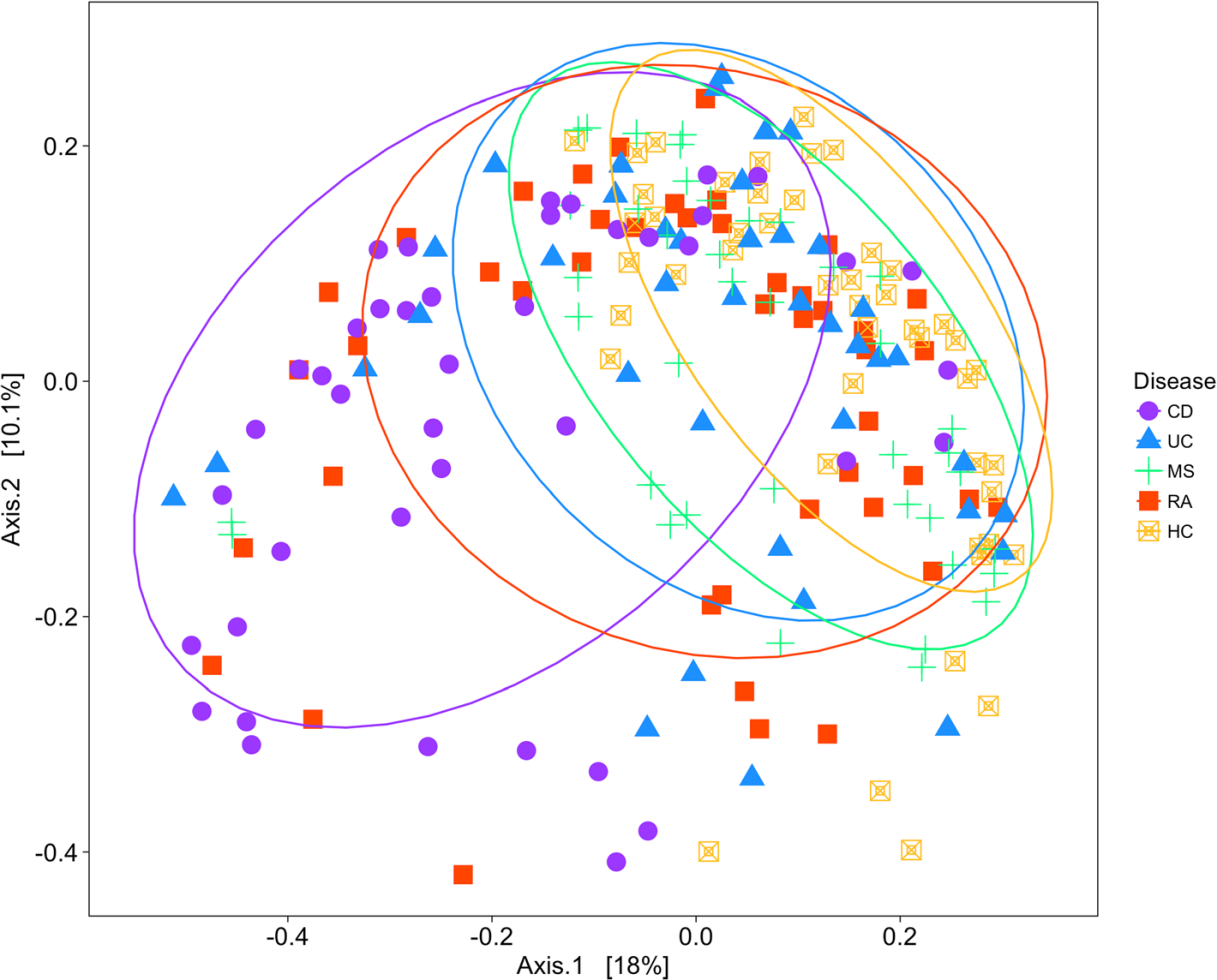
Principal coordinate analysis (PCoA) based on the overall structure of the stool microbiota in all samples. Each data point represents an individual sample. PCoA was calculated using Bray-Curtis distances with a multivariate t-distribution. Ellipses represent an 80% confidence level. Color/shape is indicative of cohort

The microbiota community structure of our cohorts was examined by principal coordinates analysis (PCoA; Fig. 1). The CD cohort had the most variability, whereas the HC cohort showed the least variability. Varying degrees of overlap (or separation) were observed for the cohort clusters. PERMANOVA analysis (Additional file 1: Table S1) showed significant microbial community differences for disease (pseudo *F* = 4.562, *R*^2^ = 0.0876, *p* = 0.001) and sex (pseudo *F* = 2.9913, *R*^2^ = 0.01915, *p* = 0.004), whereas age and sequencing run did not significantly affect the community structure. Biological replicates (from the same individual sampled at different time points) were highly similar (Additional file 1: Figure S1) suggesting that the gut microbiota is stably maintained within individuals. In addition, we calculated the Bray-Curtis dissimilarities of technical and biological replicates, from the same cohort or from different cohorts (Additional file 1: Figure S1). The dissimilarities between sample pairs of different relations are, in increasing order, technical replicates (median = 0.14; IQR = 0.08), biological replicates (median = 0.22; IQR = 0.08), intra-cohort samples (median = 0.45; IQR = 0.10), and inter-cohort samples (median = 0.47; IQR = 0.11). Overall, the dissimilarities were small; however, dissimilarities in specimens from different cohorts were significantly higher compared to those from the same cohort (Wilcoxon *p* < 0.001).

**Fig. 2:**
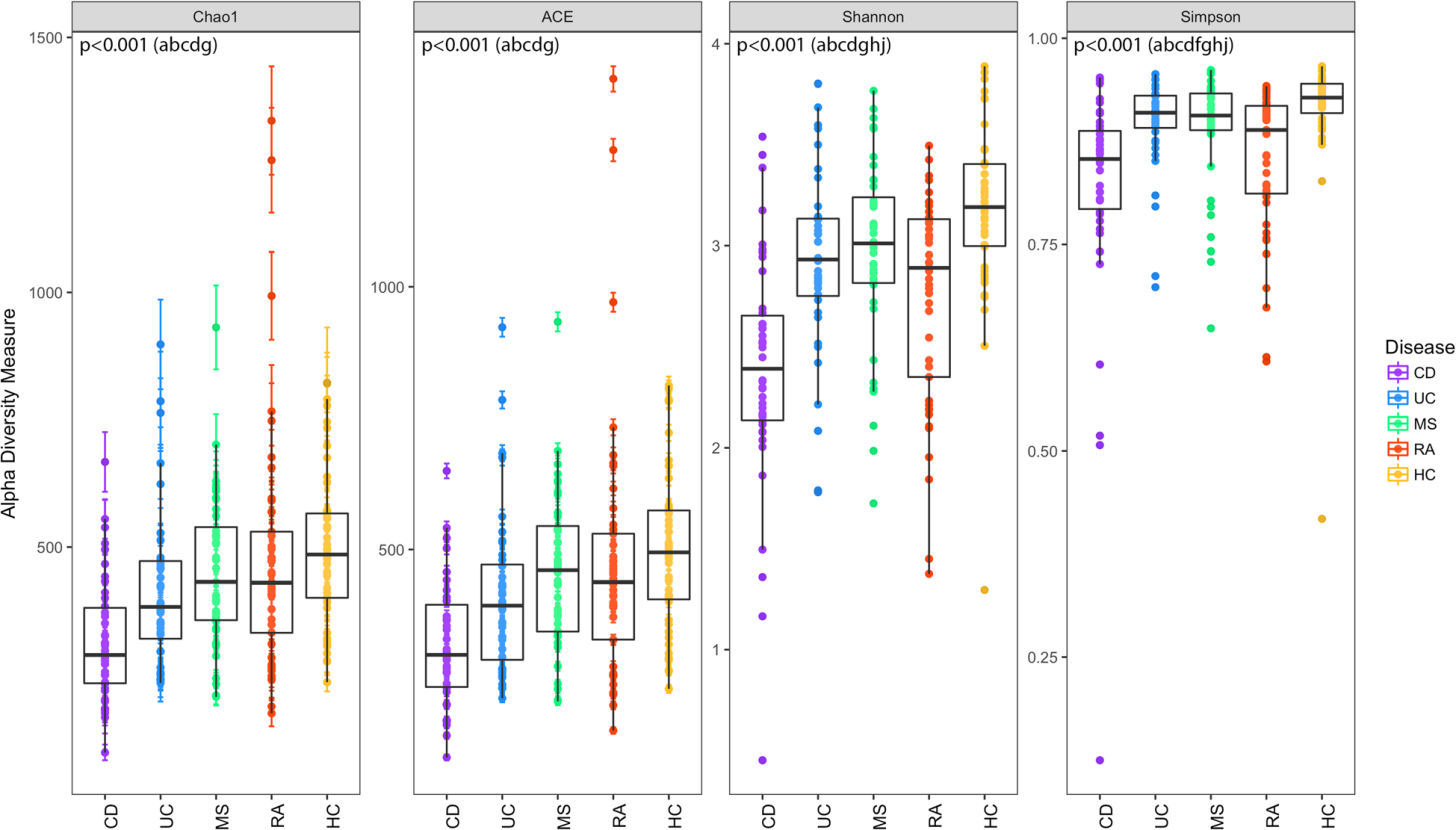
Alpha-diversity assessed by richness (Chao1, ACE) and diversity (Shannon, Simpson). Median estimates compared across cohorts using the Kruskal-Wallis test and Dunn’s post hoc tests for multiple comparisons. Boxes represent the interquartile range, lines indicate medians, and whiskers indicate the range. *p* values represent the overall FDR-corrected *p* values. ^a^CD/UC; ^b^CD/MS; ^c^CD/RA; ^d^CD/HC; ^e^UC/MS; ^f^UC/RA; ^g^UC/HC; ^h^MS/RA; ^i^MS/HC; ^j^RA/HC

Significant differences in community richness were observed across all cohorts, as estimated by Chao1 (*pFDR* < 0.001) and ACE (*pFDR* < 0.001) (Fig. 2). Richness was lowest in CD according to both estimators and was significantly different from all other cohorts with the exception of UC (via ACE). Conversely, HC had the highest richness though differences were only statistically significant compared to CD (Chao1/ACE *pFDR* < 0.001) and UC (Chao1 *pFDR* = 0.006; ACE *pFDR *= 0.01). Significant differences in diversity (Shannon, Simpson) were similarly significant across both disease cohorts and HC (*pFDR* < 0.001). Consistent with trends observed for richness, diversity was lowest in CD and highest in HC. Most cohorts demonstrated significantly different diversity with the exception of MS–UC (Shannon) and CD–RA (Simpson).

### Taxonomic composition of the gut microbiota

**Fig. 3:**
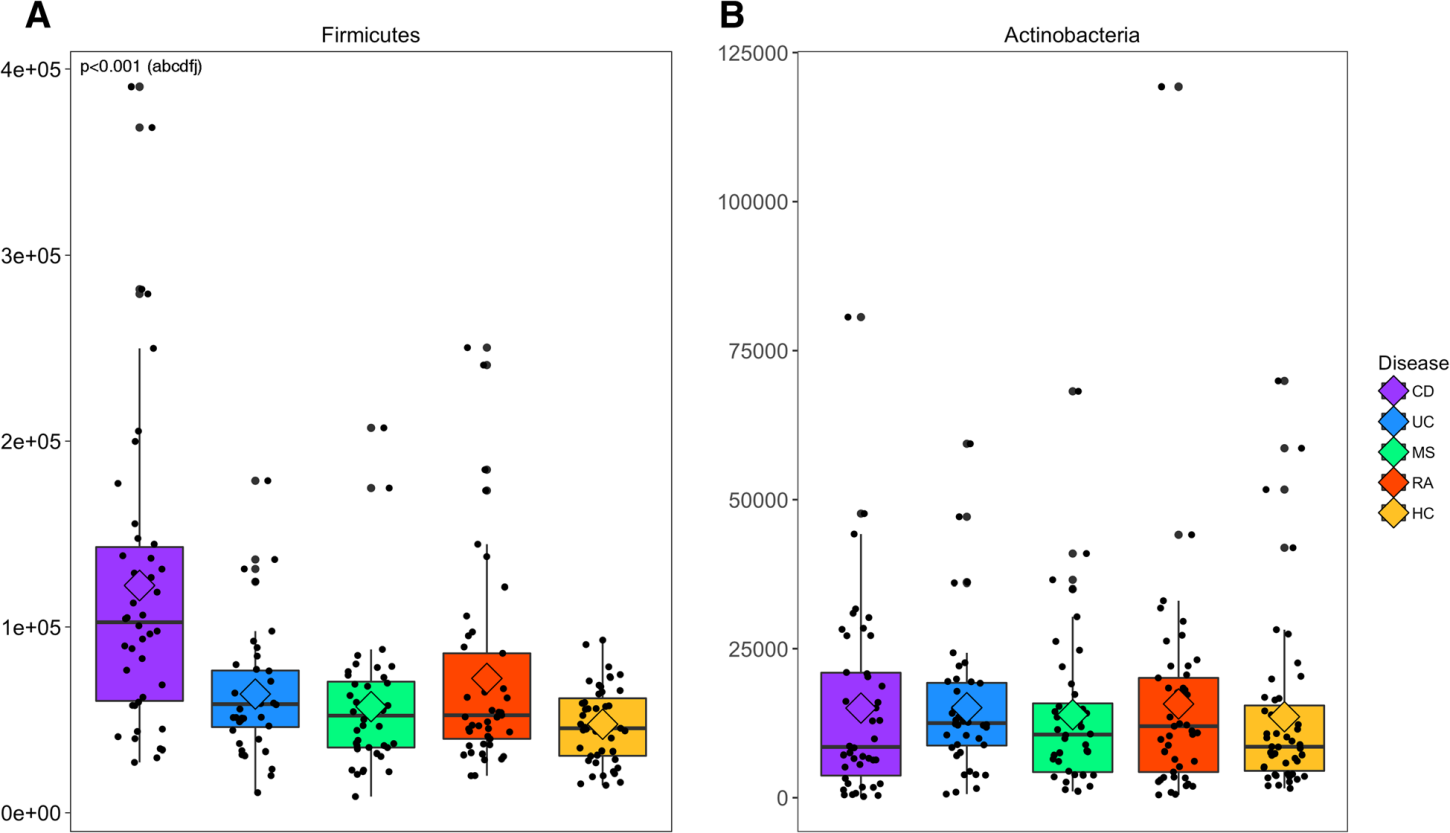
Abundance of Gram-positive phyla. Median estimates compared across cohorts using the Kruskal-Wallis test and Dunn’s post hoc tests for multiple comparisons. Boxes represent the interquartile range, lines indicate medians, diamond indicates the mean, and whiskers indicate the range. *p* values represent the overall FDR-corrected *p* values. ^a^CD/UC; ^b^CD/MS; ^c^CD/RA; ^d^CD/HC; ^e^UC/MS; ^f^UC/RA; ^g^UC/HC; ^h^MS/RA; ^i^MS/HC; ^j^RA/HC

Numerous taxonomic differences were observed between each disease cohort versus HC (Fig. 3 and Table 2). At the phylum level, the Firmicutes were significantly highest in CD and lowest in HC (overall *pFDR *< 0.001). *Actinomyces*, *Eggerthella*, *Clostridium III*, *Faecalicoccus*, and *Streptococcus* were significantly higher in all disease cohorts relative to HC, whereas the opposite was observed with *Gemmiger*, *Lachnospira*, and *Sporobacter*. Numerous other taxa also differed significantly in only one IMID compared to HC. In particular, taxa higher in disease include *Blautia* and *Intestinibacter* in CD, *Bifidobacterium* in UC, and unclassified Clostridiales incertae sedis XIII and *Erysipelotrichaceae* in MS. *Asaccharobacter*, *Clostridium* IV, *Coprococcus*, *Ruminococcus* (*Lachnospiraceae*), and *Oscillibacter* were lower in CD, *Dialister* was lower in MS, and *Roseburia* was lower in RA. Significant differences in taxon abundances were also observed in two (e.g., *Rothia*; CD and RA) or three (e.g., *Gemella*; CD, UC, and RA) diseases relative to HC. Interestingly, certain specific taxa were significantly higher in some diseases and lower in others relative to HC. For example, *Anaerofustis* was higher in UC and MS and lower in RA.

**Table 2:**
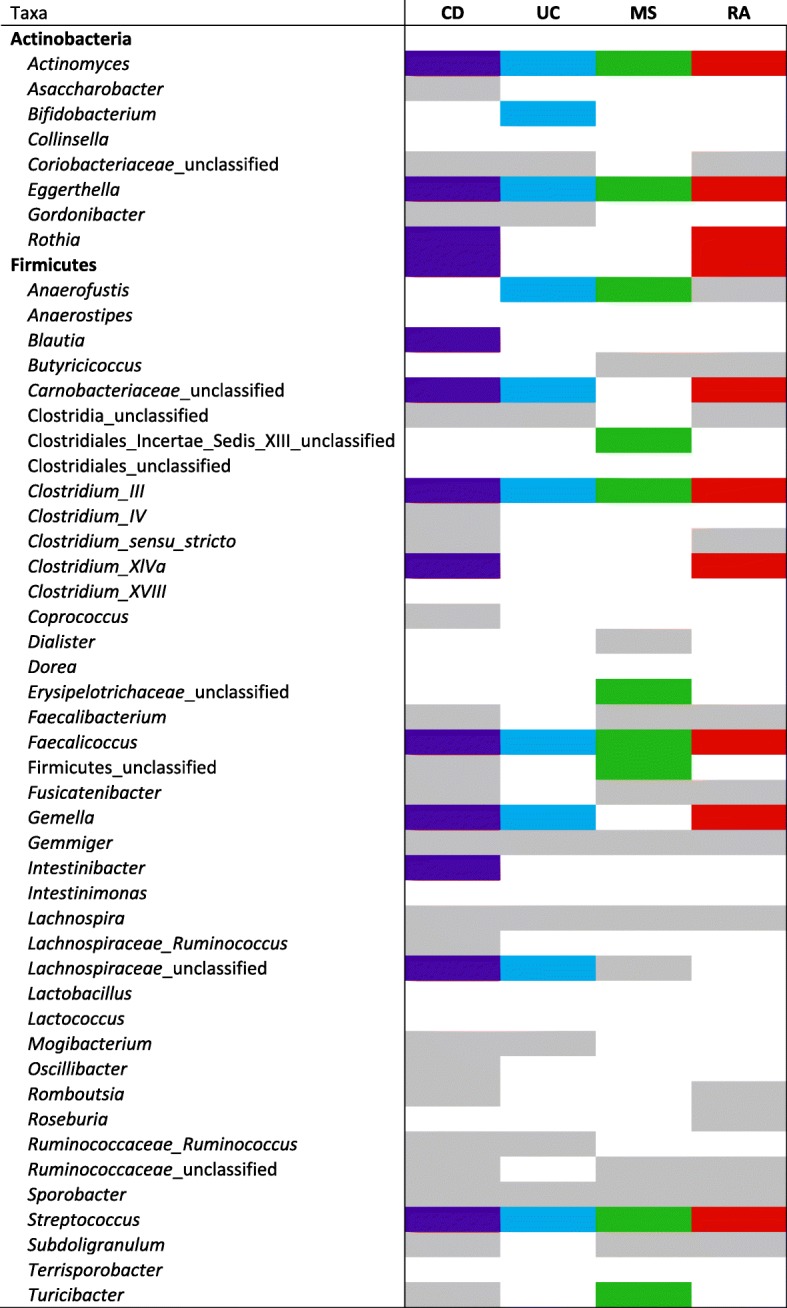
Abundant^†^ taxa in IMID microbiota relative to HC. Presence of solid color is indicative of significantly higher abundance (color) or lower abundance (gray) compared to HC

†Taxa with median abundance > 2. Taxa unable to be classified to the genus level were classified to the nearest higher taxonomic rank. Statistics were performed using the nonparametric Kruskal-Wallis test and Dunn’s post hoc tests for multiple comparisons, with FDR correction. Differences considered significant at *p* < 0.05.

Many taxonomic differences were also apparent between disease cohorts (Additional file 1: Table S2). Analogous to trends observed via PCoA, many taxa were significantly higher or lower in CD relative to other diseases. Fewer taxonomic differences were observed between MS and UC or RA, and even less between UC and RA. The abundance of *Faecalibacterium* spp*.* for example was statistically different among most disease comparisons with the exception of MS versus RA. The abundance of other genera such as *Collinsella*, *Lactobacillus*, and *Subdoligranulum* were highly similar across diseases. Taxonomic biomarkers of each cohort were identified by LEfSe analysis (Additional file 1: Figure S2). While several taxonomic biomarkers were identified, those with the largest effect size include *Blautia* (CD), *Bifidobacterium* (UC), *Ruminococcus* (*Ruminococcaceae* and *Lachnospiraceae*; MS), *Streptococcus* (RA), and (HC).

### Model performance of machine learning classifiers

To evaluate the ability of the microbiota to classify subjects to their cohort, we trained classifiers for each pair of cohorts using a supervised random forest machine learning approach. Due to reduced Gram-negative phyla in the study population, we focused on presenting data from Gram-positive phyla in the main text. For the feature classifiers, we chose to evaluate both OTUs and genera since multiple OTUs may be assigned to the same genus but have different classification abilities. Model assessments were reported as BA and AUC values (Table 3). The OTU and genus classifiers performed comparably, with 7 and 4 classifiers performing better with genus and OTU features, respectively. To further evaluate the capacity of Gram-positive phyla to classify the cohorts, we repeated the same analyses using data with all detected phyla. The overall classifier performance of the Gram-positive dataset was highly similar to that of classifiers that included both Gram-positive and Gram-negative data (Additional file 1: Table S3). Given the lower dissimilarity between the two-month same-subject biological replicates, we also evaluated the performance of predicting the cohort of samples from the second time point as a baseline. The performance was high with AUCs ranging between 0.93 and 0.99 (Additional file 1: Table S4). Similar results in classification were reported using data from all phyla (Additional file 1: Table S3).

**Table 3:**
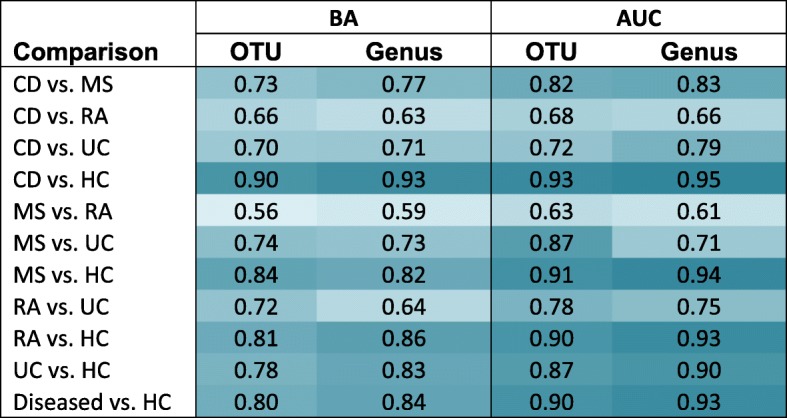
Model performance of the binary classifiers shown in balanced accuracy (BA) and area under ROC curve (AUC) indices using Gram-positive phyla

Rows represent pairs of cohorts in alphabetical order with “diseased” showing the classifier for all disease cohorts (i.e., CD, UC, MS, and RA) versus HCs. Columns represent BA and AUC indices when using either OTUs or genera as features. Performance levels are indicated relatively by a white (low) and blue (high) color scale.

The classification performance for each disease cohort versus HC in decreasing order was CD, MS, RA, and UC. The classifiers for CD versus HC had the best performance with an AUC = 0.93 and 0.95 for OTU and genus features, respectively. For UC versus HC, our classifiers reported an AUC = 0.87 and 0.90 for OTU and genus, respectively. Multi-class classification of all cohorts did not result in a satisfactory performance (BA = 0.69 and 0.67 for OTU and genus, respectively). In contrast, classification of each disease cohort versus HC had good and comparable performances with balanced sampling (AUC = 0.90 and 0.93 for OTU and genus classifiers, respectively).

### Feature importance of the classifiers

**Fig. 4:**
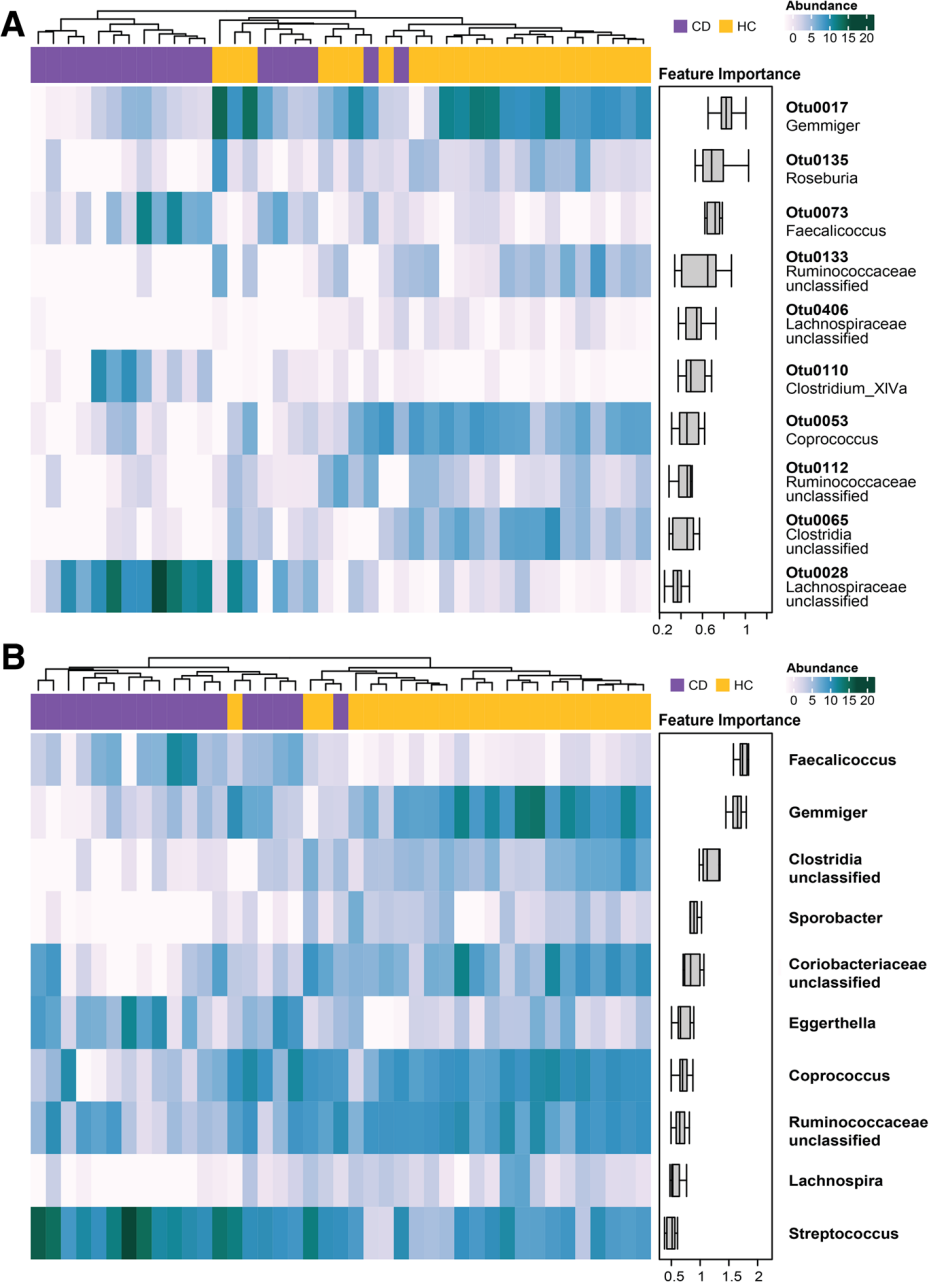
Feature importance from random forest classifiers for CD versus HCs in addition to feature abundance. Results from OTU and genus classifiers are shown in figures **a** and **b**, respectively. The corresponding genera of OTU features were labeled for the ease of interpretation. Each heatmap displays the abundance of the top ten features (rows) in samples (columns) according to the machine learning classifiers. The column bar colors represent the categories of the samples. Feature importance is shown on the right, and features are ordered in decreasing importance from top to bottom according to the mean decrease in Gini index

We also evaluated feature importance of all pairwise classifiers for Gram-negative phyla and all phyla (Additional file 1: Figures S3 and S4, respectively). The OTU and genus-level classifiers for CD versus HC are shown in Fig. 4. As previously mentioned, classifiers from different taxonomic levels (i.e., OTU, genus) can have different amounts of information content. Only one of the three *Roseburia*-associated OTUs (Otu0135) was a top feature for classifying CD versus HC, with higher abundance in HC. Conversely, the genus *Roseburia* was not found to be a top feature when using the genus-level information. The genera consistently ranking in the top three for feature importance for both classifiers included *Gemmiger* and *Faecalicoccus*, having higher abundance in HC and CD, respectively. The classifier for all disease cohorts combined versus the HC cohort identified *Faecalicoccus* as the most important feature. When examining feature importance across all binary classifiers, a common subset of features frequently ranked in the top five (Additional file 1: Tables S5 and S6). The important genera consistently found in both OTU-based and genus-based classifiers included *Anaerofustis*, *Faecalicoccus*, *Gemmiger*, *Eggerthella*, and *Faecalibacterium* whereas unclassified *Lachnospiraceae* and *Roseburia* were only of top importance based on OTU classifiers.

## Discussion

To our knowledge, this work represents the first characterization of the gut microbiota across several IMID that preferentially affect different body systems (i.e., the gastrointestinal tract, central nervous system, and synovium). This study aimed to determine patterns of gut microbiota dysbiosis relative to health and to other IMID. The results of this study are twofold. First, we found important differences in the stool microbial profile in IMID relative to health using differential abundance analyses and machine learning using the random forest algorithm. In doing so, we were able to identify consistent patterns of dysbiosis between multiple IMID in addition to dysbiosis characteristics, including taxonomic biomarkers that associate with a particular disease or group of diseases. Second, we showed how microbial populations vary between different IMIDs.

We observed a substantially lower than expected abundance of Gram-negative taxa across each of our cohorts. Accordingly, we applied our bioinformatics analysis methodologies to comparable published sequence data to validate the suitability of our samples for this investigation (Additional file 2). Thus, while the main focus of our investigation was on Gram-positive bacteria, we also evaluated the data in its entirety (e.g., Gram-positive and Gram-negative; Additional file 2). We show comparable differences in alpha and beta diversity, as well as taxonomic differences and machine learning classification performance. It is well recognized that many important gut microbial pathogens such as the *Enterobacteriaceae* are Gram-negative microorganisms [36]. However, it is equally well known that the beneficial effect or lack of anti-inflammatory Gram-positive microorganisms is imperative to host health. Out of an abundance of caution, we decided to restrict our analysis to the Gram-positive microorganisms. While it is regrettable that our dataset precluded the analysis of Gram-negative microorganisms, our findings from the Gram-positive microorganisms show that they are significantly and predictably altered depending on disease state and that specific taxa can act as biomarkers for these diseases.

Consistent with the findings of other studies investigating the gut microbiota in IMID [16, 37–39], our analysis of microbial diversity (Fig. 2) found the gut microbial diversity in IMID to be significantly lower in all IMID relative to HC according to both Shannon and Simpson indices. Interestingly, differences in diversity were also observed between disease cohorts. For example, significant differences in microbial diversity were most often observed when comparing CD and RA against the other IMIDs. While no other microbiome study to date has jointly analyzed IMIDs that target different body organs, studies have assumed “similar shifts.” For example, *Ruminococcus gnavus* was significantly higher in the gut microbiota of spondyloarthritis patients; the authors suggested that their observation is consistent with the association of *R. gnavus* and IBD [40]. As expected based on previous reports [41–43], we observed significant differences in species richness and diversity between HC and IBD (CD and UC). These studies also indicate that richness and diversity in IBD are affected by disease activity and phenotype. Prior studies have reported lower richness in RA compared to controls [16, 44], whereas others have reported no difference [45]. Although we observed decreasing trends, these differences did not reach statistical significance. Chen et al. [16] reported that lower richness in RA correlated with an elevated body mass index, rheumatoid factor levels, and disease duration while treatment regimens including methotrexate and hydrochloroquinone correlated with higher species richness (and diversity). An earlier report has shown that relapsing-remitting MS patients with active disease tended to have lower richness than those in remission [37]. We also found lower richness in MS; however, we could not differentiate whether disease activity impacted upon this finding.

We have identified several genera and OTUs consistently or individually disproportionate in abundance in IMID relative to HC or other IMIDs (Table 2, Additional file 1: Table S2). Taxonomic biomarkers of disease have previously been identified in IMID including the presence of *Escherichia coli* and absence of *Faecalibacterium prausnitzii* in CD [41]. Of importance are the taxa consistently overabundant in IMID relative to HC, which suggests they play an antagonistic role in inflammatory disease initiation and/or perpetuation and thus serve as a taxonomic biomarker for several IMIDs. For example, *Actinomyces* and *Eggerthella* spp., both of the Actinobacteria phylum, have been implicated in inflammatory conditions [46, 47], despite historical classification as commensal microorganisms [48, 49]. The implication of *E. lenta* in inflammatory diseases and other conditions has been reported in numerous studies: this microorganism was shown to be higher in type II diabetes [50], in a Japanese cohort of RRMS patients [51], and in an RA cohort [16]; *E. lenta* has also been implicated in CD bacteremia subsequent to ileocecal resection and other disseminated infections [52]. *Actinomyces* has also been reported to be higher in RA [47] and non-inflamed UC mucosa [42]. Interestingly, CD patients following therapy with enteral nutrition or anti-TNFα antibodies had decreased *Actinomyces* spp. whereas *Lactococcus* and *Roseburia* were increased [53]. In our random forest classifiers for CD compared to HC, a *Roseburia* OTU was identified as an important feature (higher abundance in HC), suggesting its importance for health. Accordingly, the genus *Roseburia* [54] was reported to be a potential marker of health due to its butyrate-producing and anti-inflammatory properties. Taxa within the Firmicutes phylum, including *Clostridium* III, *Faecalicoccus*, and *Streptococcus* were also higher in IMID compared to HC. *Streptococcus* spp. in particular have been reported to be higher in numerous IMID [16, 51], and evidence shows that particular species can influence disease severity in animal models of disease [55]. Due to their pathogenic potential, candidate species such as *S. thermophilus* and *S. pneumoniae* have been assessed in the context of inflammatory conditions. In contrast, there is limited literature pertaining to the genus *Faecalicoccus*, which may be ascribed to its taxonomic classification, as reports from 2014 and 2015 indicate that it was recently classified as a novel genus [56, 57]. The consistently higher abundance of these microorganisms in IMIDs relative to HC warrants further study to determine the possible role these microorganisms in the etiology of IMID.

Several taxa were higher or lower abundance in a subset of IMIDs compared to HC. As an example, we observed *Rothia* [58], an opportunistic pathogen, to be more abundant in CD and RA. *Rothia* spp. have been shown to be higher in IMID including CD [59] and ankylosing spondylitis [60]. *Gemella*, a common inhabitant of mucosal membranes, was similarly higher in abundance in CD, RA, and UC. Gevers et al. [59] reported *G. moribillum* to be present in higher abundance in CD compared to controls. Interestingly, a recent experimental colitis study has reported that *Gemella*, in addition to *Ruminococcus* and *Blautia,* were overrepresented in the arthritis and colitis group versus arthritis only, at 41 days post-induction [61]. There are numerous environmental and genetic risk factors that are known to influence IMID. Thus, it is not surprising that some taxa demonstrate varying degrees of differences in abundance compared to HC. Of note, taxa such as *Anaerofustis* were in higher abundance in UC and MS, but in lower abundance in RA compared to HC. Though previously isolated from human stool [62], currently there is no evidence linking *Anaerofustis* to disease.

Conversely, we have reported some genera to be preferentially underrepresented in IMID, many of which are consistent with the findings of other studies and suggestive of a potential protective effect against disease. We observed *Gemmiger* to be significantly lower in all IMIDs. *Gemmiger* is consistently enriched in HC, specifically in the context of comparisons with IBD, non-alcoholic steatohepatitis, *Clostridium difficile* infection, and colorectal cancer [63, 64]. In addition, *Gemmiger* spp. are included in some probiotic compositions [65]. Attributed to their reduced abundance, studies indicate that *Lachnospira* [66] may have a protective role in inflammatory conditions. We reported numerous genera (and OTUs) to be uniquely underrepresented in IMID compared to HC and further observed significant differences of specific microorganisms between IMIDs. While we did detect some commonalities indicating the members of these genera may share similar pathogenic mechanisms, it is highly likely that some microorganisms exhibit differential effects on distinct IMID. For example, *Bifidobacterium* was higher in UC compared to HC and has similarly been described in UC patients [67], which is interesting given the probiotic nature of the microorganism. Thus, we propose that the depletion of particular anti-inflammatory microorganisms may be disease-specific (perhaps related to treatment) and encourage further investigation into their effect on gut homeostasis.

We also observed many taxa that were significantly different between IMIDs (Additional file 1: Table S2). Considering IMIDs included in this study preferentially target different body organs, it is not surprising that we detected differences. Moreover, while similar genetic and environmental factors are involved in IMID etiopathogenesis, there are risk factors that are unique to disease, for example, Epstein-Barr virus [68] or appendectomy in CD [69]. Even within IBD (i.e., CD and UC), it is well recognized that differences exist between the two gastrointestinal diseases [42].

On a sample-level, the Bray-Curtis dissimilarity that we observed between technical replicates was comparable to previous reports [28]. In particular, our results revealed higher variability between, rather than within, individuals. This is consistent with studies that described the stability of the microbiome within individuals [70], and microbiome-based identifiability, where individuals can be robustly identified with their microbiota data over time [71].

To augment our differential abundance analysis, we conducted machine learning using a random forest approach to conduct binary classification of diseases versus HC using OTU or genus abundance as features. Machine learning approaches successfully used in recent related studies have incorporated a variety of features including taxonomic [72], functional [72], and k-mer-based [64] classification schemes. In our study, we chose to evaluate the feature importance of OTU and genus classifiers, since they can in theory differ in their ability to classify disease state versus HC. While the overall performances are comparable between OTU and genus models, the feature importance of *Roseburia*, for example, was highlighted in the OTU model rather than in the genus model comparing CD to HC. The ability to classify subjects at a higher taxonomic level (e.g., genus) may potentially be compromised due to differences in abundance levels among OTUs of a genus. On the other hand, subtle differential abundance levels at a lower taxonomic level (e.g., OTU) may cumulatively contribute to a stronger differential abundance signal at the genus level.

For the purpose of this study, we have shown that despite the known importance in pathogenicity of Gram-negative bacteria, machine learning classification between pairs of cohorts using only data from Gram-positive microorganisms resulted in similar performances to using the complete dataset (i.e., Gram-positive and Gram-negative). Further, our CD versus HC classifiers performed slightly better than previously reported classifiers for IBD (CD and UC combined) versus HC [64, 73]. In contrast, our multi-disease classifier performed poorly, which is expected considering similar poor performances were observed for our binary classifiers between different diseases, such as MS versus RA, CD versus RA, and CD versus UC. These results suggest that the similarities in gut microbiota composition between diseases make their classification difficult. This notion is reinforced by the reduced inter-disease variability (versus HC) observed in the PCoA plot (Fig. 1). Consistently, the high performances in diseased versus HC models highlights the degree of commonality in taxonomic abundance among IMIDs compared to HC. The classification results demonstrate the capacity of classifying HC versus the four disease cohorts from our study (as one cohort, as well as individual cohorts) using 16S rRNA gene amplicon gut microbiota sequencing data.

One particular strength of our study is the investigation of the gut microbiota composition from several IMIDs that preferentially target different body organs. This allows us to identify putative taxonomic biomarkers common to varying IMIDs. The limitation of our study is certainly the unusually low abundance of Gram-negative microorganisms relative to datasets analyzed in similar studies [59, 70]. Upon an extensive investigation through wet-laboratory work, data exploration, and literature review, we believe the low abundance of Gram-negative microorganisms was likely a result of sub-optimal storage conditions such as multiple freeze-thaw cycles or long-term storage [74]. We addressed this limitation by directing our analyses on the Gram-positives and by testing our machine learning classifiers against the full dataset. Differential abundance testing and diversity analyses were also completed on the full dataset (see Additional file 2). The low abundance of some taxa (e.g., Bacteroidetes and Proteobacteria) did not noticeably reduce the ability of our machine learning classifiers to assign diseased and healthy samples. These study findings reveal that microbiota characterization can be performed using “imperfect” datasets, which may be further overcome through the use of robust machine learning approaches. Other limitations of this study include the use of stool rather than mucosa (which houses distinct and more immunologically related microorganisms) and the targeted evaluation of the bacterial component of the microbiome rather than including microorganisms such as fungi or viruses.

Importantly, our study was also limited by the lack of certain patient-related data. These include factors related to disease (e.g., phenotype, activity, and duration) and prior or current treatment and lifestyle factors (e.g., diet and smoking). Additional disease-specific or lifestyle factors may also have influenced the microbiome composition. Even if they were known, due to the small sample size, our study could not account for the biases of potential confounding factors. In a case where an unobserved influential factor is unequally distributed between cohorts, the statistical power and model performance may be overestimated as a result of reporting additional taxonomic markers driven by the confounding factor. On the other hand, when such an unobserved factor is not unequally distributed between cohorts, the power and model performance may be lowered. While these should be taken into consideration when interpreting results, we note that many taxa identified were corroborated by those reported in the literature, confirming our study’s capacity to detect taxonomic biomarkers between disease cohorts and HCs. Validations with other comparable and larger cohorts will need to be performed to ensure these taxonomic biomarkers are consistently identified while also adjusting for confounding factors. In subsequent work comparing the gut microbiome from different IMIDs, it will be especially important to have large enough cohorts of participants within disease groups who have active disease and cohorts with inactive disease to account for the role of an active systemic immune response on the gut microbiome. Similarly, it will be important to have sample sizes sufficient to allow for comparisons across different treatments. There remains a dearth of data as to the impact of biological therapy and other immunomodulating therapies on the gut microbiota.

The interplay between the gut microbiota and the systemic immune responses will be a very important area for study in contrasting different IMIDs. Aberrant host immune responses are well recognized in IMID yet incompletely understood. The role of the microbiota in the involvement with the development of several immune cells including Th1, Th2, Th17, and Treg cells is well recognized [75]. There is much interest in linking gut microbiota changes to the host immune response in IMID. Organisms such as *F*. *prausnitzii* [76] and *Clostridium* spp. [77] have a role in directing Treg cells and their response. Since Treg cells are well recognized to be involved in all IMIDs, the importance of these microorganisms may be in their impact on Treg cell abnormalities. Our results showed differential abundance among IMID for these microorganisms, though more experiments are required to evaluate the potential links to differences in pathogenesis among IMID reported previously [78]. As more studies like ours emerge that describe gut microbiota changes across various IMID, further research will be necessary to determine the interactions between the taxa that are altered in IMID and their specific immune responses.

## Conclusions

In summary, this study presents a comprehensive analysis of the stool microbiota composition in several IMIDs. We conclude that the composition of the gut microbiota is altered in CD, UC, MS, and RA, especially relating to varying degrees of gut dysbiosis that were evident. Moreover, we also show that within IMIDs, several microorganisms demonstrated significant compositional differences. For example, the gut microbial communities in UC and RA were most similar among IMIDs; whereas the gut microbiota of CD was most different from other IMIDs as well as HC. We have uncovered several microorganisms that are consistently higher (or lower) in IMID relative to HC using differential abundance testing and machine learning, suggesting that there may be common microbial taxonomic biomarkers for IMID. Further research into these microorganisms and their associated functions within the host are needed in order to establish any causality in disease pathogenesis and future therapeutic potential.

## Additional files

Additional file 1: **Table S1.** PERMANOVA analysis used to assess microbial community structure differences. **Table S2.** Median abundance of abundant taxa in IMID and HC microbiota. **Table S3.** Model performance of the binary classifiers trained on data using all phyla. **Table S4.** Model performance of the binary classifiers trained on data from the first biological replicate and predicted on the second biological replicate. **Table S5.** Importance of top 5 features in any of the OTU classifiers using Gram-positive phyla data. **Table S6.** Importance of top 5 features in any of the genus classifiers using Gram-positive phyla data. **Figure S1.** Sample distances shown in Bray-Curtis dissimilarity and principal coordinate analysis (PCoA) plot. **Figure S2.** Taxa identified as discriminating features for IMIDs versus HCs. **Figure S3.** Feature importance from pair-wise machine learning classifiers using all Phyla. **Figure S4.** Feature importance from pair-wise machine learning classifiers using Gram-positive phyla data. (DOCX 10707 kb)

Additional file 2: Comparison of Gram positive only microbiota versus total microbiota. (XLSX 120 kb)

## Abbreviations

ACE: Abundance-based coverage estimator
AUC: Area under the receiver operating characteristic curve
BA: Balanced accuracy
CD: Crohn’s disease
HC: Healthy control
IBD: Inflammatory bowel disease
IMID: Immune-mediated inflammatory disease
IQR: Interquartile range
MS: Multiple sclerosis
OOB: Out of bag
OTU: Operational taxonomic unit
PCoA: Principal coordinate analysis
PERMANOVA: Permutational analysis of variance
RA: Rheumatoid arthritis
rRNA: Ribosomal RNA
UC: Ulcerative colitis
