# Supplementary material for: A comparative study of the gut microbiota in immune-mediated inflammatory diseases—does a common dysbiosis exist?
Source: Microbiome. 2018 Dec 13;6:221. doi: 10.1186/s40168-018-0603-4 (PMC6292067; doi:10.1186/s40168-018-0603-4)

Supplemental material for: **A comparative study of the gut microbiota in immune-mediated inflammatory diseases – Does a common dysbiosis exist?**

(Forbes et al.)

Table S1 PERMANOVA analysis used to assess microbial community structure differences

Table S2 Median abundance of abundant† taxa in IMID and HC microbiota

Table S3 Model performance of the binary classifiers trained on data using all phyla

Table S4 Model performance of the binary classifiers trained on data from the first biological replicate and predicted on the second biological replicate

Table S5 Importance of top 5 features in any of the OTU classifiers using Gram-positive phyla data

Table S6 Importance of top 5 features in any of the genus classifiers using Gram-positive phyla data

Figure S1 Sample distances shown in Bray-Curtis dissimilarity and principal coordinate analysis (PCoA) plot

Figure S2 Taxa identified as discriminating features for IMIDs versus HCs

Figure S3 Feature Importance from pair-wise machine learning classifiers using all Phyla

Figure S4 Feature Importance from pair-wise machine learning classifiers using Gram-positive phyla data

Supplemental File 1

**Table S1. PERMANOVA analysis used to assess microbial community structure differences.** Statistically significant differences are shown in bold.

|  | F-model | R2 | Pr(>F) |
| --- | --- | --- | --- |
| Disease | 4.562 | 0.0876 | **0.001** |
| Age | 1.7874 | 0.01144 | 0.058 |
| Sex | 2.9913 | 0.01915 | **0.004** |
| Run | 0.9429 | 0.02414 | 0.553 |

**Table S2. Median abundance of abundant† taxa in IMID and HC microbiota.**

| **Taxa** | **CD** | **UC** | **MS** | **RA** | **HC** | **Overall pFDR** | **Pairwise pFDR** |
| --- | --- | --- | --- | --- | --- | --- | --- |
| **Actinobacteria** | 8472.71 | 12483.20 | 10557.63 | 11964.91 | 8511.93 |  |  |
| *Actinomyces* | 56.33 | 47.58 | 53.20 | 67.42 | 24.99 | *** | dfghij |
| *Asaccharobacter* | 15.97 | 226.56 | 269.75 | 274.99 | 348.27 | *** | abcd |
| *Bifidobacterium* | 1926.23 | 6221.10 | 3411.39 | 4010.15 | 2291.54 |  | ag |
| *Collinsella* | 1368.86 | 3573.20 | 2901.30 | 3345.47 | 3287.02 |  |  |
| *Coriobacteriaceae*_unclassified | 14.06 | 91.47 | 241.10 | 58.94 | 319.53 | *** | abcdghj |
| *Eggerthella* | 242.49 | 111.50 | 222.10 | 119.00 | 16.46 | *** | dgij |
| *Gordonibacter* | 10.57 | 34.86 | 80.25 | 40.57 | 66.71 | ** | bdegh |
| *Rothia* | 7.23 | 2.18 | 1.96 | 5.36 | 1.72 | *** | abdfhj |
| **Firmicutes** | 102530.70 | 58393.57 | 52200.91 | 52413.42 | 45389.70 | *** | abcdfj |
| *Anaerofustis* | 0.49 | 12.09 | 11.07 | 5.65 | 1.78 | *** | abcghij |
| *Anaerostipes* | 2268.73 | 1532.79 | 1903.23 | 2040.22 | 1227.36 |  |  |
| *Blautia* | 29266.20 | 15806.64 | 13704.44 | 13010.46 | 14454.37 | *** | abcd |
| *Butyricicoccus* | 30.54 | 49.66 | 3.77 | 20.06 | 102.70 | *** | befhij |
| *Carnobacteriaceae*_unclassified | 27.04 | 13.22 | 5.61 | 9.07 | 4.27 | *** | abcdegj |
| Clostridia_unclassified | 1.62 | 48.34 | 172.59 | 28.43 | 99.61 | *** | abcdghj |
| Clostridiales_Incertae_Sedis_XIII_unclassified | 0.00 | 26.61 | 61.26 | 18.74 | 31.83 | *** | abcdehi |
| Clostridiales_unclassified | 2893.62 | 3443.33 | 4126.82 | 4182.58 | 3464.29 |  |  |
| *Clostridium_III* | 1.01 | 2.47 | 19.39 | 12.33 | 0.00 | *** | bdgij |
| *Clostridium_IV* | 65.86 | 286.54 | 211.19 | 262.73 | 322.19 | ** | abcd |
| *Clostridium_sensu_stricto* | 34.89 | 103.31 | 497.89 | 62.73 | 256.33 | *** | bdehj |
| *Clostridium_XlVa* | 663.18 | 143.55 | 384.18 | 250.28 | 74.37 | *** | adefij |
| *Clostridium_XVIII* | 651.69 | 1287.22 | 975.76 | 587.09 | 867.76 |  |  |
| *Coprococcus* | 36.92 | 777.67 | 606.65 | 564.38 | 937.01 | ** | abcd |
| *Dialister* | 4.51 | 5.43 | 1.08 | 2.96 | 4.65 | * | bei |
| *Dorea* | 2331.80 | 1303.04 | 1229.85 | 1331.11 | 1387.46 |  | c |
| *Erysipelotrichaceae*_unclassified | 3.46 | 10.55 | 14.12 | 6.22 | 5.79 |  | bji |
| *Faecalibacterium* | 61.72 | 2602.16 | 523.33 | 882.07 | 2748.36 | *** | abcdefij |
| *Faecalicoccus* | 80.74 | 31.70 | 26.64 | 40.51 | 3.12 | *** | dgij |
| Firmicutes_unclassified | 7.91 | 52.86 | 141.96 | 87.01 | 66.33 | *** | abcdehi |
| *Fusicatenibacter* | 429.08 | 693.18 | 263.81 | 324.05 | 913.53 | * | deij |
| *Gemella* | 13.98 | 5.05 | 3.16 | 6.78 | 1.45 | *** | abcdeghj |
| *Gemmiger* | 10.87 | 71.51 | 175.27 | 281.76 | 741.38 | *** | bcdegij |
| *Intestinibacter* | 177.36 | 56.51 | 126.15 | 40.22 | 51.54 | * | cd |
| *Intestinimonas* | 35.03 | 69.73 | 111.52 | 120.29 | 116.36 |  |  |
| *Lachnospira* | 0.00 | 3.17 | 1.20 | 3.07 | 9.05 | *** | acdgij |
| *Lachnospiraceae*_*Ruminococcus* | 311.49 | 706.10 | 1725.81 | 552.96 | 874.73 | ** | bdeh |
| *Lachnospiraceae*_unclassified | 1599.48 | 822.90 | 558.07 | 675.78 | 573.11 | *** | abcdeg |
| *Lactobacillus* | 61.92 | 17.34 | 18.97 | 30.73 | 29.72 |  |  |
| *Lactococcus* | 41.09 | 42.28 | 67.76 | 25.93 | 35.42 |  |  |
| *Mogibacterium* | 5.13 | 4.92 | 7.26 | 8.29 | 30.40 | ** | cdg |
| *Oscillibacter* | 2.53 | 6.16 | 7.91 | 16.56 | 11.61 | ** | bcd |
| *Romboutsia* | 83.83 | 997.74 | 751.80 | 330.60 | 1078.18 | *** | abcdfhj |
| *Roseburia* | 636.21 | 1206.82 | 437.39 | 252.89 | 966.18 |  | fhj |
| *Ruminococcaceae*_*Ruminococcus* | 31.22 | 142.70 | 629.82 | 888.77 | 960.31 | *** | abcdeg |
| *Ruminococcaceae*_unclassified | 93.42 | 820.30 | 408.13 | 366.88 | 823.01 | *** | adefij |
| *Sporobacter* | 0.00 | 1.01 | 3.66 | 3.46 | 21.52 | *** | abcdgij |
| *Streptococcus* | 2462.20 | 1078.06 | 1502.50 | 2860.54 | 675.44 | *** | abdfghij |
| *Subdoligranulum* | 17.59 | 29.89 | 42.16 | 26.20 | 133.33 | ** | dij |
| *Terrisporobacter* | 23.41 | 52.01 | 13.64 | 9.29 | 20.14 |  |  |
| *Turicibacter* | 3.16 | 86.87 | 98.66 | 19.07 | 43.12 | *** | abcdfhi |

†Taxa with median abundance >2. Taxa unable to be classified to the genus level were classified according to a higher taxonomic rank. Statistics were performed using the nonparametric Kruskal-Wallis test and Dunn’s post-hoc tests for multiple comparisons, with FDR correction; *p<0.05, **p<0.01, ***p<0.001. Shading corresponds to median abundance of taxa. ^a^CD/UC; ^b^CD/MS; ^c^CD/RA; ^d^CD/HC; ^e^UC/MS; ^f^UC/RA; ^g^UC/HC; ^h^MS/RA; ^i^MS/HC; ^j^RA/HC.

**Table S3.** **Model performance of the binary classifiers trained on data using all phyla.**

|  | **OOB performance** | | | |  |  | **Biological replicates** | | | |
| --- | --- | --- | --- | --- | --- | --- | --- | --- | --- | --- |
| **Comparison** | **BA** | | **AUC** | |  |  | **BA** | | **AUC** | |
|  | **OTU** | **Genus** | **OTU** | **Genus** |  | **Comparison** | **OTU** | **Genus** | **OTU** | **Genus** |
| CD vs. MS | 0.74 | 0.77 | 0.82 | 0.83 |  | CD vs. MS | 0.94 | 0.91 | 0.98 | 0.97 |
| CD vs. RA | 0.63 | 0.63 | 0.70 | 0.68 |  | CD vs. RA | 0.91 | 0.81 | 0.96 | 0.91 |
| CD vs. UC | 0.70 | 0.70 | 0.74 | 0.76 |  | CD vs. UC | 0.86 | 0.87 | 0.94 | 0.94 |
| CD vs. HC | 0.90 | 0.91 | 0.94 | 0.95 |  | CD vs. HC | 0.95 | 0.92 | 0.99 | 0.97 |
| MS vs. RA | 0.57 | 0.59 | 0.61 | 0.61 |  | MS vs. RA | 0.82 | 0.81 | 0.94 | 0.94 |
| MS vs. UC | 0.71 | 0.67 | 0.83 | 0.67 |  | MS vs. UC | 0.90 | 0.92 | 0.98 | 0.97 |
| MS vs. HC | 0.82 | 0.83 | 0.90 | 0.93 |  | MS vs. HC | 0.89 | 0.91 | 0.98 | 0.97 |
| RA vs. UC | 0.70 | 0.73 | 0.77 | 0.80 |  | RA vs. UC | 0.89 | 0.89 | 0.97 | 0.96 |
| RA vs. HC | 0.81 | 0.83 | 0.89 | 0.91 |  | RA vs. HC | 0.95 | 0.86 | 0.99 | 0.95 |
| UC vs. HC | 0.77 | 0.79 | 0.89 | 0.89 |  | UC vs. HC | 0.94 | 0.84 | 0.99 | 0.95 |
| Diseased vs. HC | 0.78 | 0.82 | 0.90 | 0.92 |  | Diseased vs. HC | 0.87 | 0.87 | 0.96 | 0.94 |

**Table S4.** **Model performance of the binary classifiers trained on data from the first biological replicate and predicted on the second biological replicate.**

|  | **BA** | | **AUC** | |
| --- | --- | --- | --- | --- |
| **Comparison** | **OTU** | **Genus** | **OTU** | **Genus** |
| CD vs. MS | 0.93 | 0.89 | 0.98 | 0.98 |
| CD vs. RA | 0.92 | 0.84 | 0.97 | 0.93 |
| CD vs. UC | 0.86 | 0.86 | 0.93 | 0.94 |
| CD vs. HC | 0.95 | 0.92 | 0.99 | 0.97 |
| MS vs. RA | 0.84 | 0.84 | 0.94 | 0.94 |
| MS vs. UC | 0.93 | 0.93 | 0.99 | 0.99 |
| MS vs. HC | 0.89 | 0.93 | 0.99 | 0.97 |
| RA vs. UC | 0.84 | 0.86 | 0.97 | 0.94 |
| RA vs. HC | 0.91 | 0.88 | 0.98 | 0.97 |
| UC vs. HC | 0.94 | 0.83 | 0.98 | 0.95 |
| Diseased vs. HC | 0.88 | 0.87 | 0.96 | 0.94 |

**Table S5. Importance of top 5 features in any of the OTU classifiers using Gram-positive phyla data.** Feature importance is reported as the mean decrease in Gini index for the OTU classifiers. Rows are sorted by the counts of classifiers in which each OTU is in the top 5 features.

| **OTU** | **Genus** | **CD/MS** | **CD/RA** | **CD/UC** | **CD/HC** | **MS/RA** | **MS/UC** | **MS/HC** | **RA/UC** | **RA/HC** | **UC/HC** | **Diseased/HC** |
| --- | --- | --- | --- | --- | --- | --- | --- | --- | --- | --- | --- | --- |
| Otu0073 | *Faecalicoccus* | 0.06 | 0.05 | 0.05 | 0.71 | 0.05 | 0.04 | 0.60 | 0.04 | 0.57 | 0.65 | 0.80 |
| Otu0021 | *Faecalibacterium* | 0.04 | 0.08 | 0.59 | 0.20 | 0.10 | 0.84 | 0.54 | 0.44 | 0.14 | 0.04 | 0.14 |
| Otu0176 | *Anaerofustis* | 0.44 | 0.15 | 0.30 | 0.04 | 0.37 | 0.03 | 0.83 | 0.18 | 0.34 | 0.42 | 0.40 |
| Otu0406 | *Lachnospiraceae*_unclassified | 0.01 | 0.21 | 0.01 | 0.53 | 0.25 | 0.01 | 0.60 | 0.24 | 0.03 | 0.87 | 0.41 |
| Otu0017 | *Gemmiger* | 0.32 | 0.31 | 0.10 | 0.82 | 0.05 | 0.07 | 0.08 | 0.05 | 0.16 | 0.48 | 0.37 |
| Otu0135 | *Roseburia* | 0.04 | 0.02 | 0.26 | 0.76 | 0.05 | 0.08 | 0.42 | 0.27 | 0.55 | 0.10 | 0.52 |
| Otu0541 | *Faecalibacterium* | 0.02 | 0.01 | 0.34 | 0.02 | 0.00 | 0.59 | 0.20 | 0.59 | 0.11 | 0.07 | 0.03 |
| Otu0042 | *Eggerthella* | 0.03 | 0.05 | 0.06 | 0.30 | 0.04 | 0.14 | 0.29 | 0.18 | 0.35 | 0.38 | 0.45 |
| Otu0109 | *Lachnospiracea*e_unclassified | 0.03 | 0.04 | 0.43 | 0.21 | 0.06 | 0.16 | 0.11 | 0.43 | 0.33 | 0.03 | 0.17 |
| Otu0133 | *Ruminococcaceae*_unclassified | 0.14 | 0.02 | 0.18 | 0.60 | 0.05 | 0.05 | 0.23 | 0.13 | 0.47 | 0.05 | 0.37 |
| Otu0156 | *Clostridium_XlVa* | 0.15 | 0.04 | 0.03 | 0.23 | 0.41 | 0.10 | 0.05 | 0.05 | 0.55 | 0.21 | 0.26 |
| Otu0178 | *Clostridium_IV* | 0.03 | 0.06 | 0.56 | 0.03 | 0.04 | 0.48 | 0.04 | 0.25 | 0.02 | 0.18 | 0.03 |
| Otu0012 | *Ruminococcus* | 0.54 | 0.14 | 0.17 | 0.18 | 0.42 | 0.10 | 0.14 | 0.09 | 0.03 | 0.07 | 0.04 |
| Otu0025 | *Asaccharobacter* | 0.17 | 0.43 | 0.19 | 0.14 | 0.05 | 0.05 | 0.04 | 0.04 | 0.02 | 0.04 | 0.06 |
| Otu0027 | *Intestinibacter* | 0.03 | 0.33 | 0.04 | 0.04 | 0.29 | 0.04 | 0.05 | 0.24 | 0.06 | 0.12 | 0.05 |
| Otu0037 | *Staphylococcus* | 0.01 | 0.27 | 0.02 | 0.01 | 0.22 | 0.02 | 0.01 | 0.37 | 0.24 | 0.01 | 0.03 |
| Otu0065 | Clostridia_unclassified | 0.06 | 0.05 | 0.36 | 0.43 | 0.05 | 0.05 | 0.21 | 0.13 | 0.26 | 0.04 | 0.21 |
| Otu0071 | Clostridiales_Incertae_Sedis_XIII_unclassified | 0.65 | 0.30 | 0.11 | 0.24 | 0.19 | 0.35 | 0.15 | 0.06 | 0.03 | 0.04 | 0.05 |
| Otu0092 | *Intestinimonas* | 0.01 | 0.26 | 0.05 | 0.06 | 0.36 | 0.10 | 0.10 | 0.10 | 0.02 | 0.04 | 0.04 |
| Otu0097 | *Faecalibacterium* | 0.04 | 0.03 | 0.04 | 0.18 | 0.16 | 0.02 | 0.10 | 0.13 | 0.50 | 0.13 | 0.27 |
| Otu0111 | Clostridia_unclassified | 0.41 | 0.13 | 0.03 | 0.01 | 0.14 | 0.73 | 0.31 | 0.15 | 0.06 | 0.02 | 0.05 |
| Otu0169 | *Lachnospiraceae*_unclassified | 0.21 | 0.05 | 0.08 | 0.03 | 0.49 | 0.19 | 0.13 | 0.13 | 0.04 | 0.03 | 0.05 |
| Otu0212 | *Lactonifactor* | 0.42 | 0.22 | 0.04 | 0.02 | 0.05 | 0.24 | 0.60 | 0.07 | 0.27 | 0.03 | 0.17 |
| Otu0302 | *Mogibacterium* | 0.03 | 0.30 | 0.11 | 0.01 | 0.18 | 0.06 | 0.03 | 0.06 | 0.10 | 0.07 | 0.06 |
| Otu0352 | *Blautia* | 0.02 | 0.08 | 0.03 | 0.01 | 0.05 | 0.16 | 0.08 | 0.38 | 0.15 | 0.03 | 0.05 |
| Otu0475 | *Lachnospiraceae*_unclassified | 0.08 | 0.35 | 0.04 | 0.01 | 0.05 | 0.01 | 0.01 | 0.03 | 0.07 | 0.02 | 0.02 |

**Table S6. Importance of top 5 features in any of the genus classifiers using Gram-positive phyla data.** Feature importance is reported as the mean decrease in Gini index for the genus classifiers. Rows are sorted by the counts of classifiers in which each genus is in the top 5 features.

| **Genus** | **CD/MS** | **CD/RA** | **CD/UC** | **CD/HC** | **MS/RA** | **MS/UC** | **MS/HC** | **RA/UC** | **RA/HC** | **UC/HC** | **Diseased/HC** |
| --- | --- | --- | --- | --- | --- | --- | --- | --- | --- | --- | --- |
| *Anaerofustis* | 0.86 | 0.39 | 0.74 | 0.10 | 0.81 | 0.13 | 1.87 | 0.49 | 0.87 | 1.14 | 1.02 |
| *Faecalicoccus* | 0.09 | 0.14 | 0.12 | 1.77 | 0.13 | 0.13 | 1.22 | 0.14 | 1.74 | 2.02 | 2.18 |
| *Gemmiger* | 0.68 | 0.69 | 0.24 | 1.64 | 0.18 | 0.25 | 0.22 | 0.15 | 0.38 | 1.01 | 0.87 |
| Clostridia_unclassified | 1.18 | 0.61 | 0.86 | 1.17 | 0.41 | 0.24 | 0.18 | 0.13 | 0.32 | 0.19 | 0.36 |
| *Eggerthella* | 0.10 | 0.11 | 0.18 | 0.70 | 0.14 | 0.43 | 0.71 | 0.36 | 0.85 | 0.91 | 0.95 |
| *Enterococcus* | 0.05 | 0.12 | 0.06 | 0.21 | 0.21 | 0.10 | 0.85 | 0.11 | 0.34 | 0.82 | 0.77 |
| *Intestinibacter* | 0.09 | 0.71 | 0.18 | 0.13 | 0.78 | 0.13 | 0.19 | 0.61 | 0.19 | 0.32 | 0.17 |
| *Lactonifactor* | 0.89 | 0.48 | 0.09 | 0.05 | 0.14 | 0.65 | 1.54 | 0.23 | 0.80 | 0.16 | 0.58 |
| *Butyricicoccus* | 0.25 | 0.17 | 0.11 | 0.08 | 0.67 | 0.84 | 0.73 | 0.19 | 0.17 | 0.09 | 0.22 |
| Clostridiales_Incertae_Sedis_XIII_unclassified | 1.19 | 0.64 | 0.26 | 0.49 | 0.52 | 0.90 | 0.44 | 0.15 | 0.09 | 0.15 | 0.18 |
| *Clostridium_XlVa* | 0.10 | 0.21 | 0.81 | 0.35 | 0.19 | 0.58 | 0.37 | 0.58 | 0.50 | 0.14 | 0.34 |
| *Faecalibacterium* | 0.13 | 0.42 | 1.35 | 0.39 | 0.26 | 0.94 | 0.37 | 0.47 | 0.23 | 0.12 | 0.29 |
| *Ruminococcaceae*_unclassified | 0.22 | 0.30 | 0.54 | 0.66 | 0.12 | 0.64 | 0.48 | 0.61 | 0.43 | 0.11 | 0.38 |
| *Staphylococcus* | 0.05 | 0.60 | 0.05 | 0.02 | 0.53 | 0.08 | 0.05 | 0.93 | 0.64 | 0.05 | 0.11 |
| *Asaccharobacter* | 0.33 | 0.80 | 0.38 | 0.39 | 0.19 | 0.19 | 0.13 | 0.12 | 0.08 | 0.11 | 0.14 |
| *Clostridium_III* | 0.16 | 0.27 | 0.14 | 0.19 | 0.12 | 0.19 | 0.94 | 0.15 | 0.62 | 0.27 | 0.59 |
| *Clostridium_XVIII* | 0.14 | 0.19 | 0.29 | 0.10 | 0.34 | 0.16 | 0.09 | 0.63 | 0.32 | 0.16 | 0.18 |
| *Coriobacteriaceae*_unclassified | 0.27 | 0.21 | 0.30 | 0.85 | 0.25 | 0.14 | 0.14 | 0.26 | 0.52 | 0.34 | 0.48 |
| *Ruminococcus* | 0.84 | 0.15 | 0.30 | 0.17 | 0.69 | 0.21 | 0.22 | 0.20 | 0.09 | 0.11 | 0.12 |
| *Sporobacter* | 0.41 | 0.24 | 0.10 | 0.88 | 0.12 | 0.24 | 0.33 | 0.15 | 0.23 | 0.78 | 0.61 |
| *Streptococcus* | 0.12 | 0.14 | 0.35 | 0.50 | 0.31 | 0.13 | 0.19 | 0.26 | 0.85 | 0.18 | 0.50 |
| *Turicibacter* | 0.33 | 0.15 | 0.64 | 0.18 | 0.26 | 0.20 | 0.13 | 0.38 | 0.12 | 0.16 | 0.15 |

**
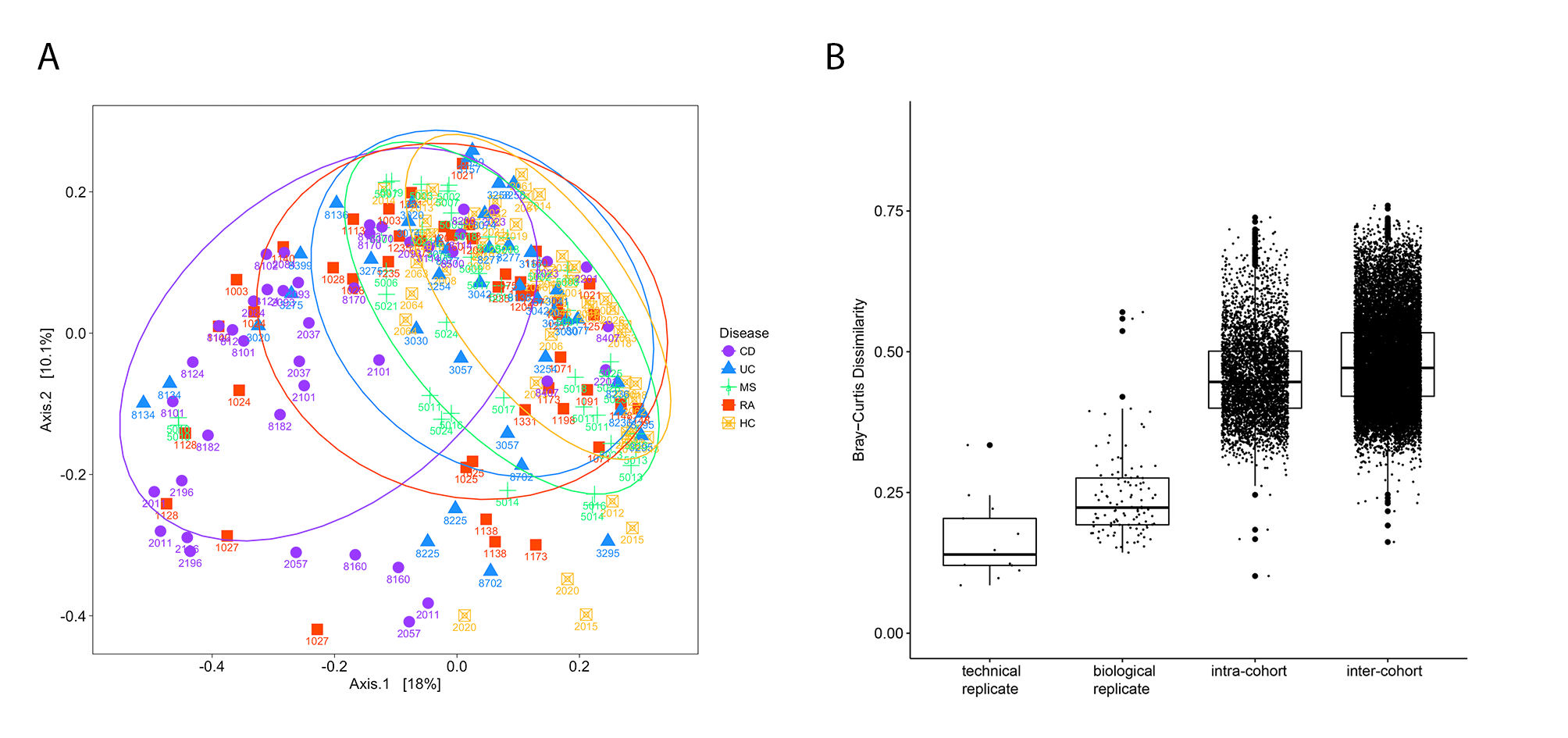
**

**Figure S1. Sample distances shown in Bray-Curtis dissimilarity and principal coordinate analysis (PCoA) plot**. (A). PCoA based on the overall structure of the stool microbiota in all samples. Each data point represents an individual sample. PCoA was calculated using Bray-Curtis distances with a multivariate t-distribution. Ellipses represent an 80% confidence level. Colour/shape is indicative of cohort. Data points are labelled by sample ID. (B) Boxplot distribution of Bray-Curtis dissimilarity between sample pairs of different relations after normalization. The y-axis represents the Bray-Curtis distance, and the x-axis represents relations between pairs of samples, including technical replicates, biological replicates, samples within the same cohort and samples from different cohorts.

**

**

**Figure S2. Taxa identified as discriminating features for IMIDs versus HCs.** Features identified by LEfSe analysis. Significance determined by Kruskal-Wallis test and post-hoc Wilcoxon test. Features considered significant at p<0.05. Taxa shown include those with an LDA score >3.

**Figure S3.**  **Feature Importance from pair-wise machine learning classifiers using all Phyla**


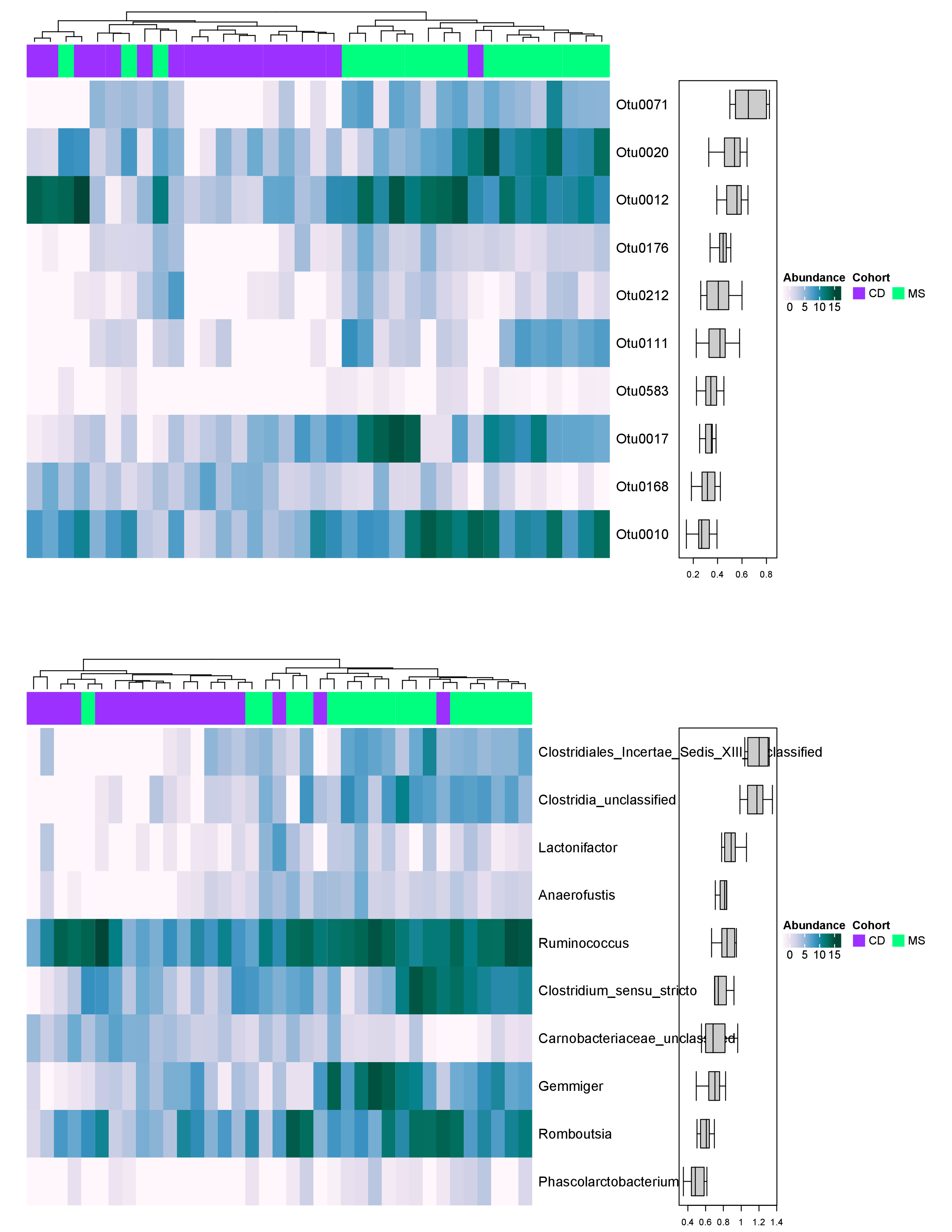

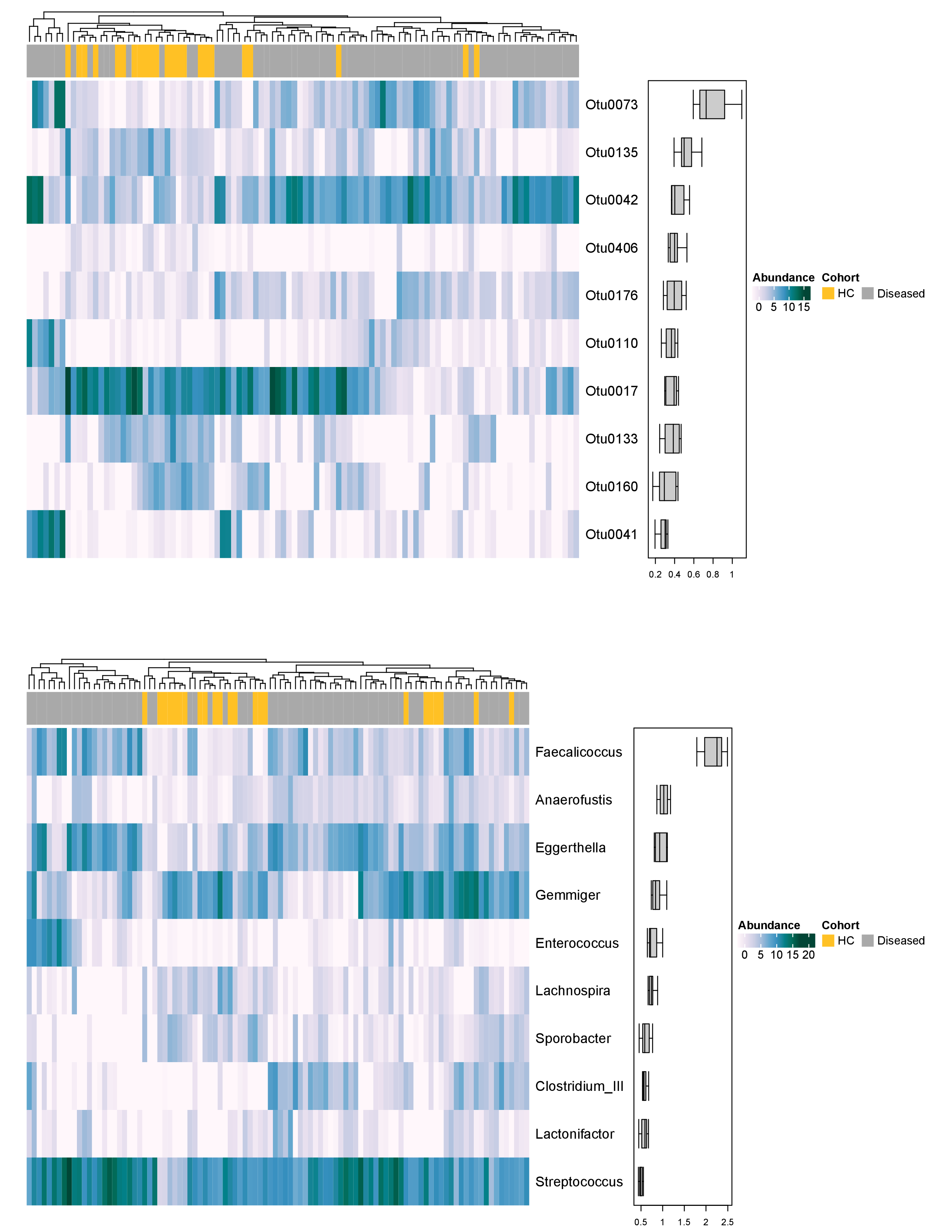

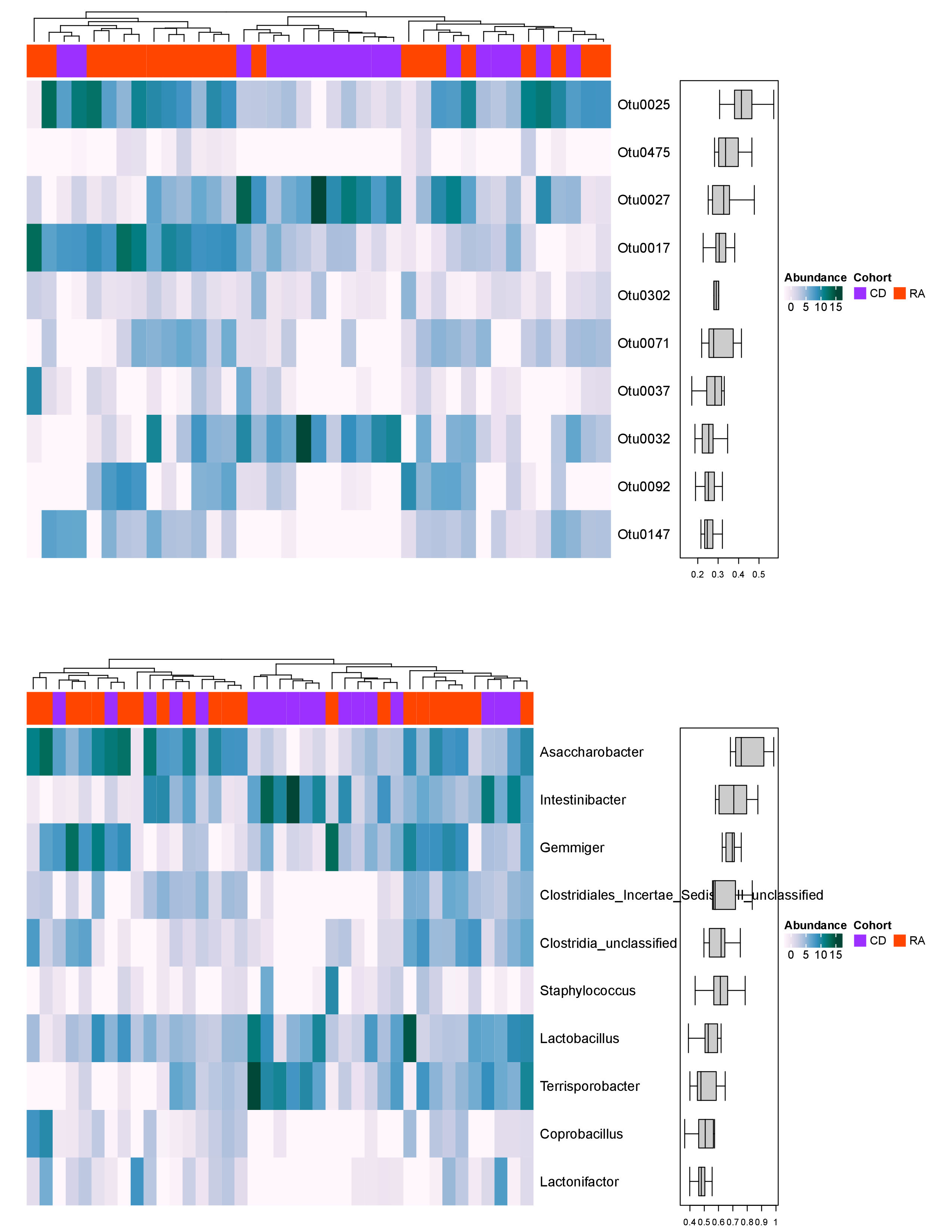

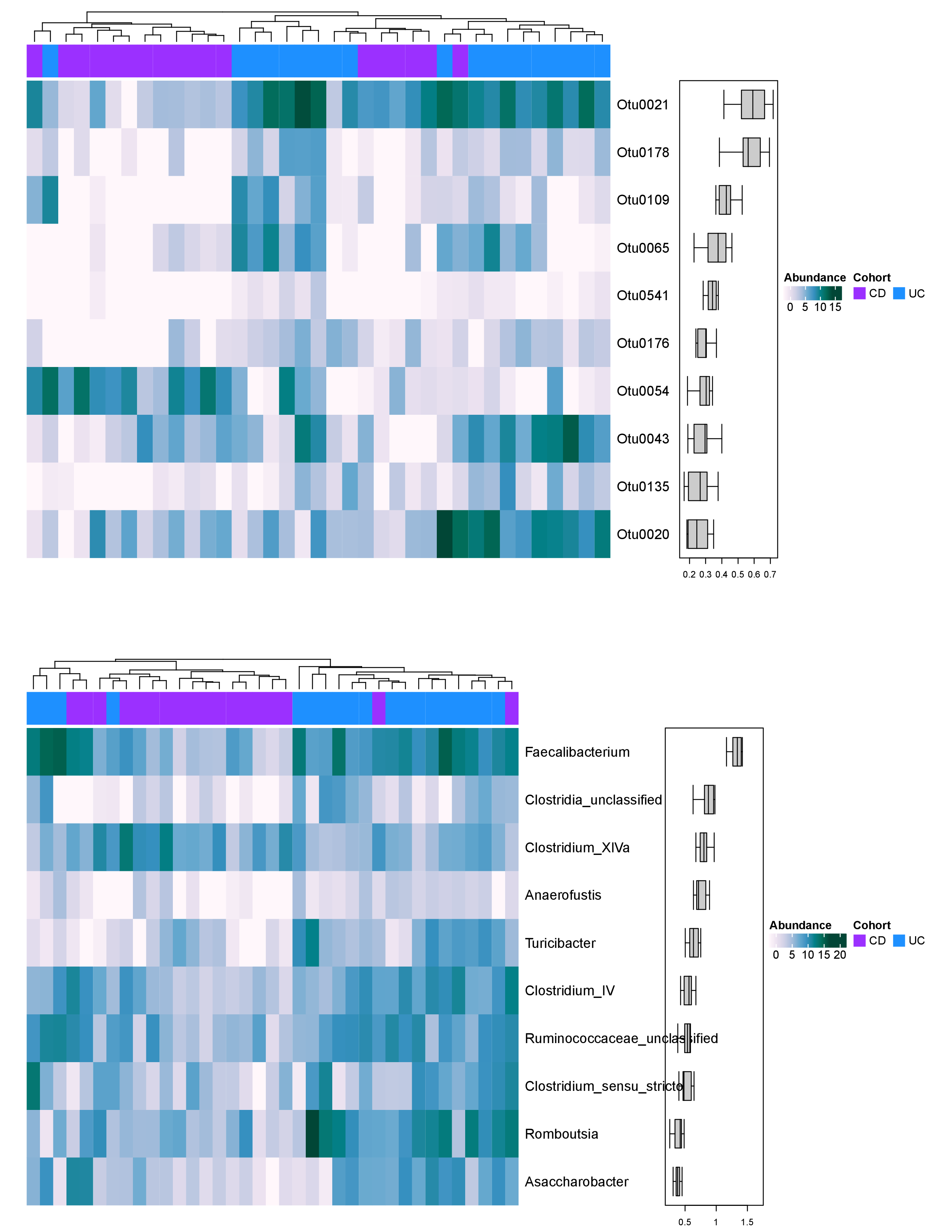

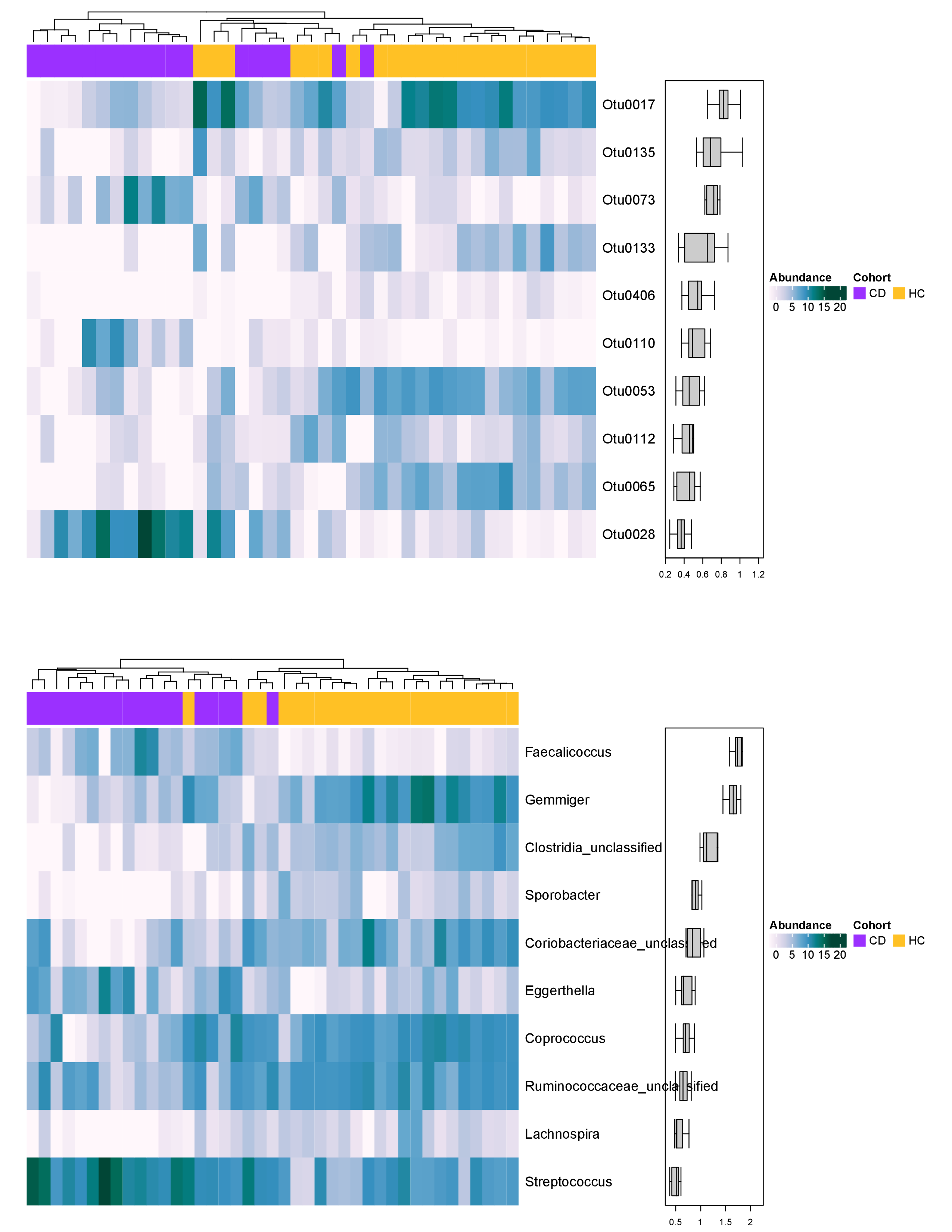

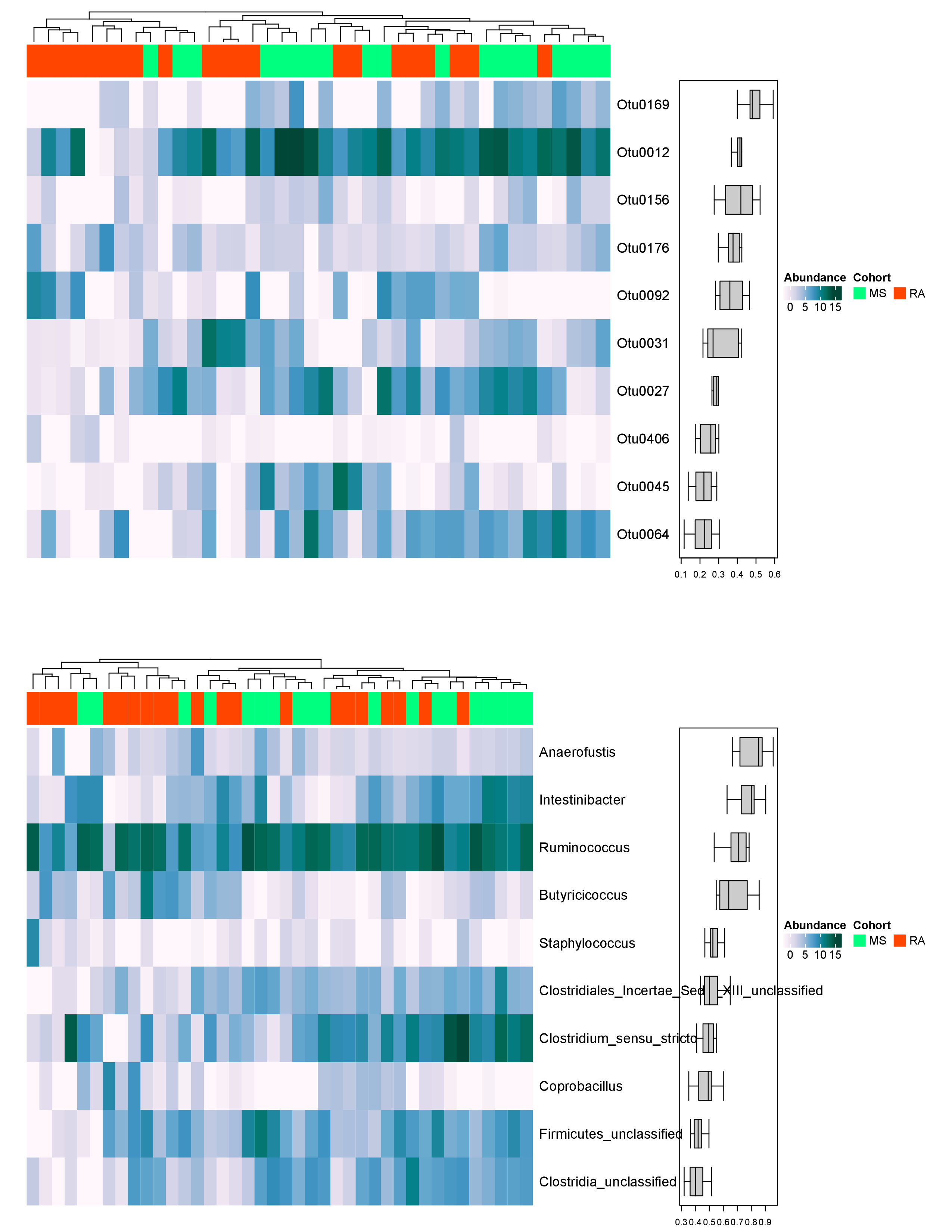

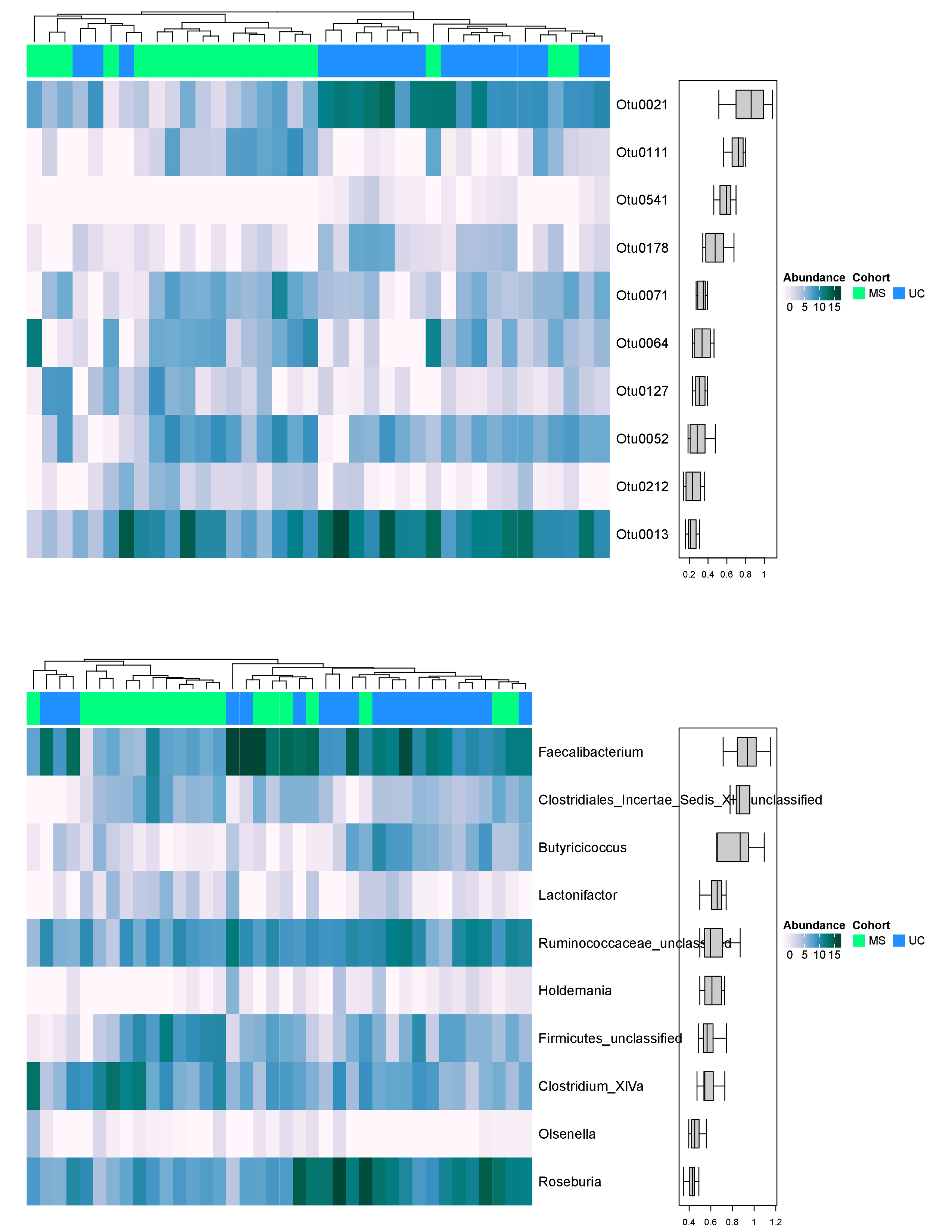

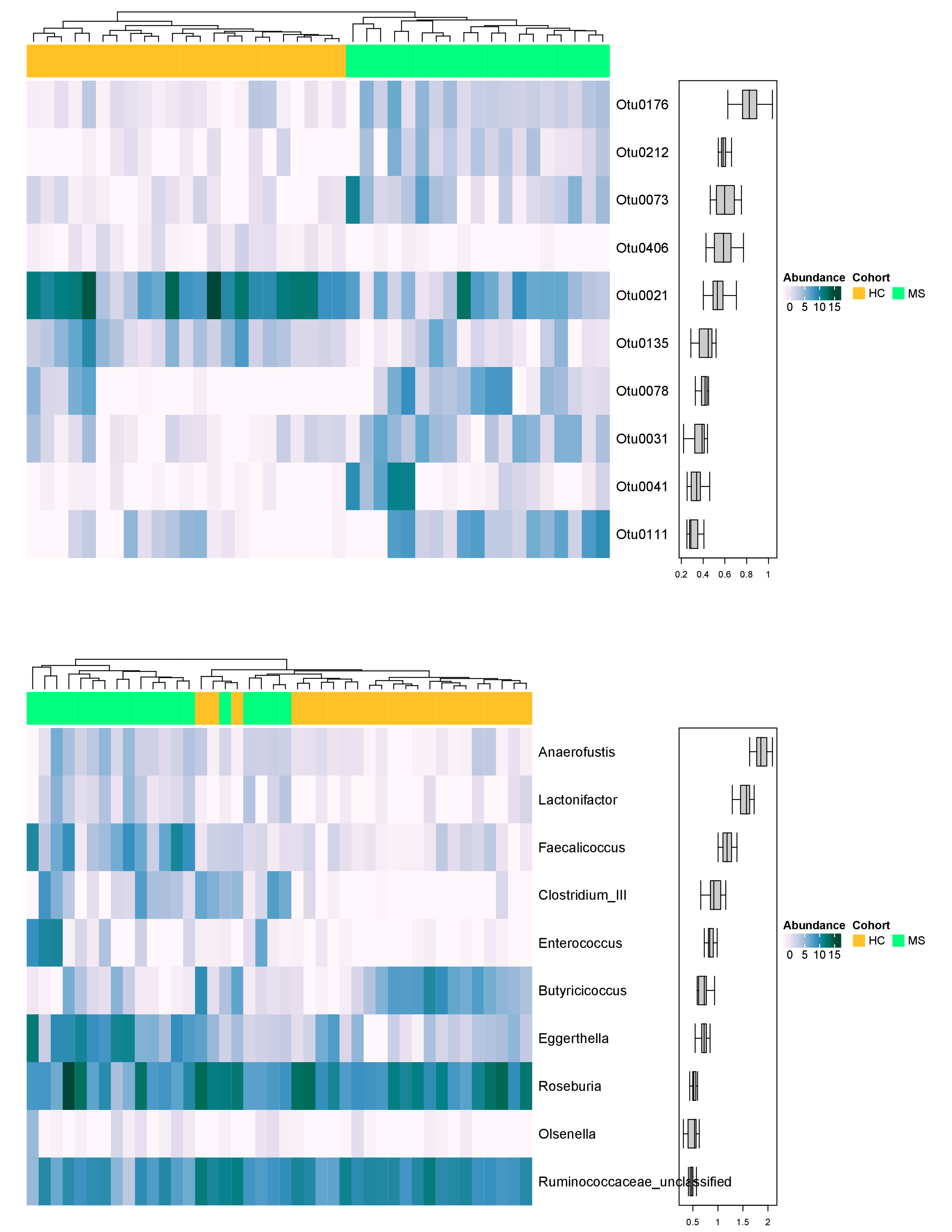

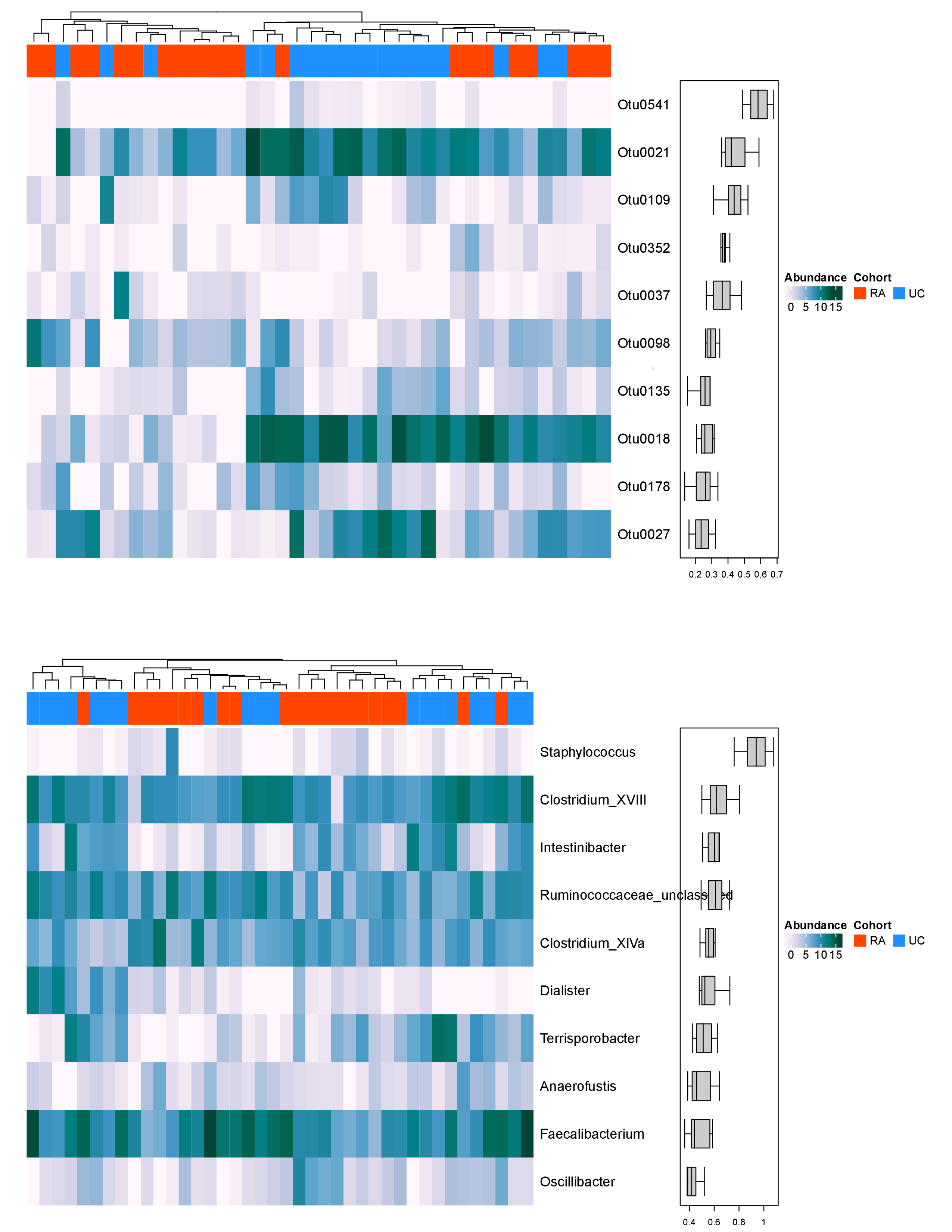

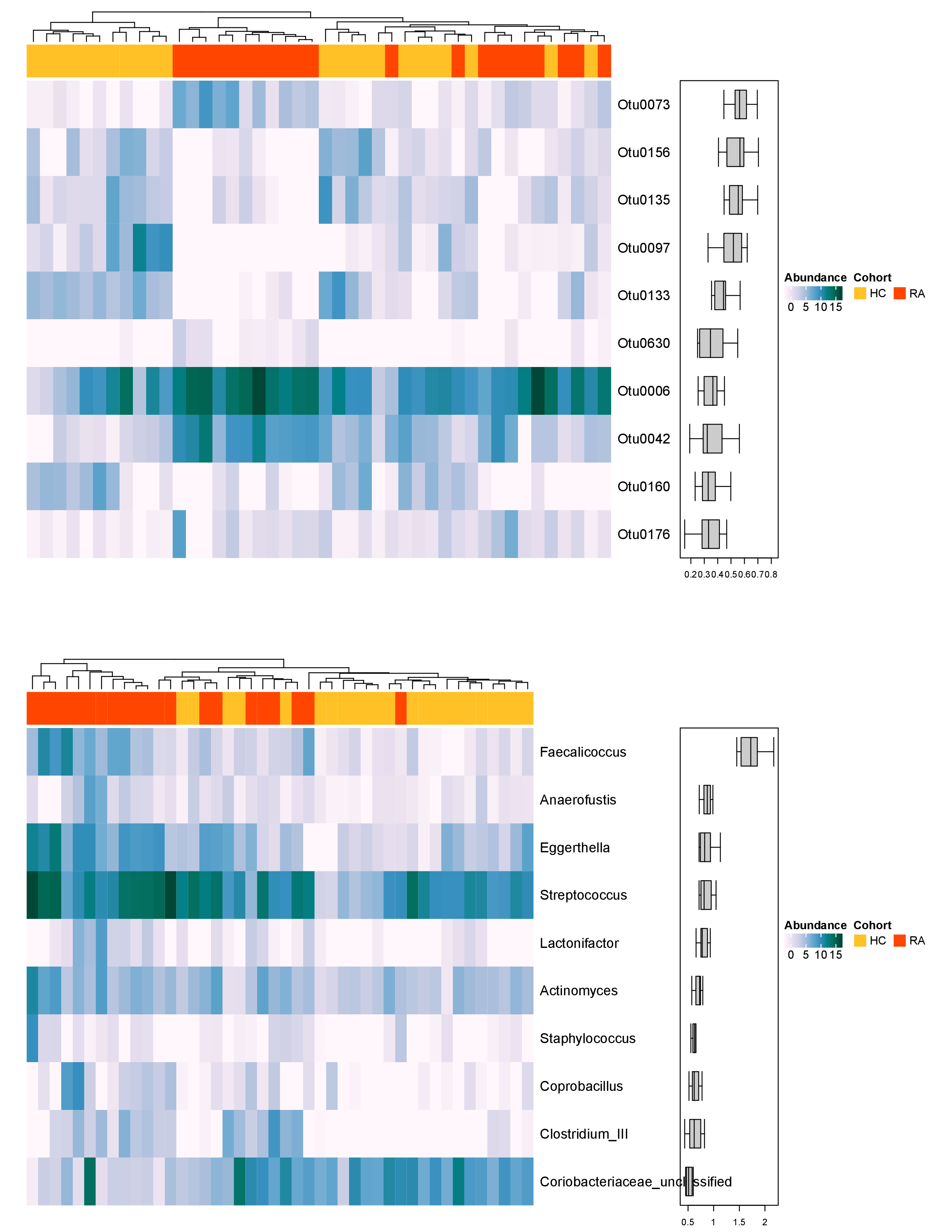

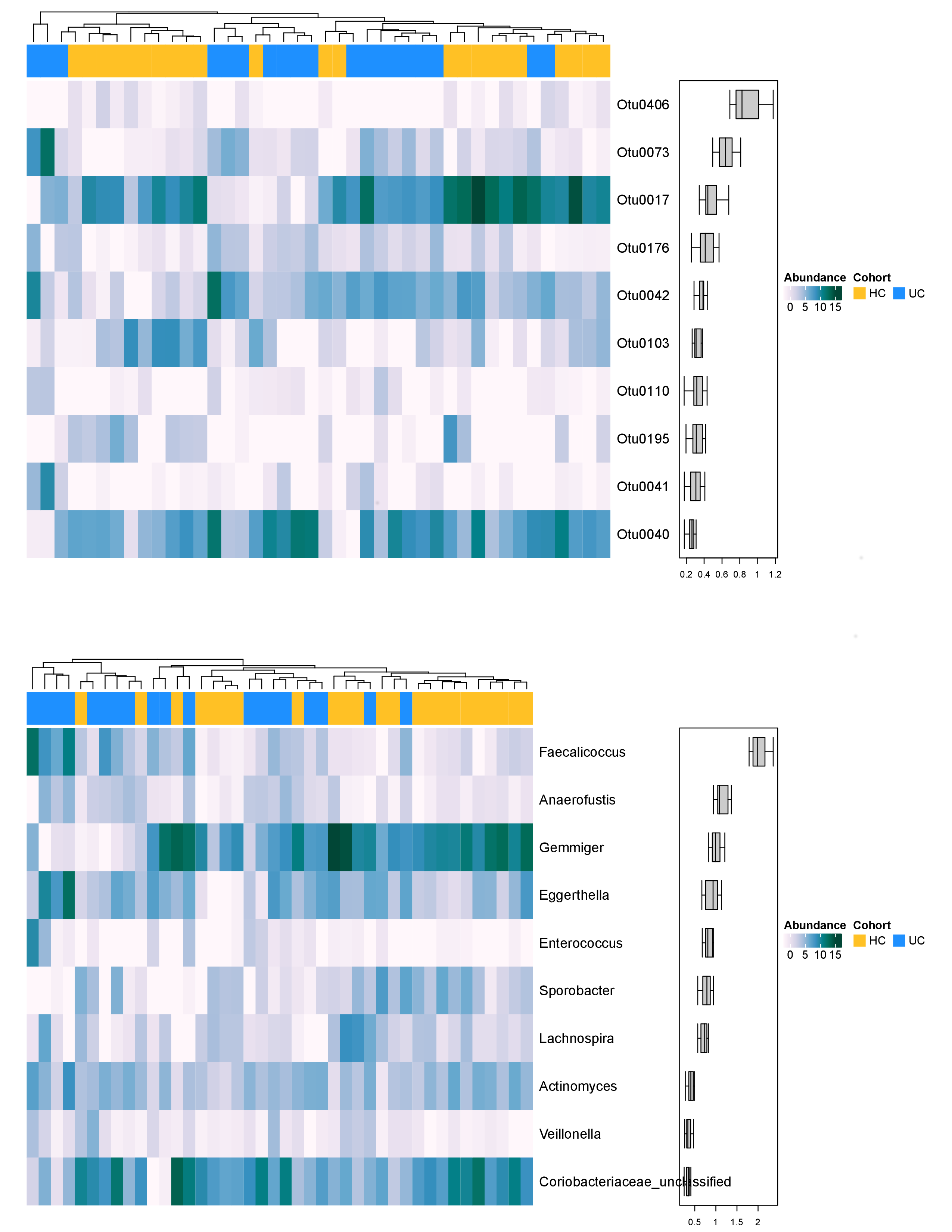


**Figure S4. Feature Importance from pair-wise machine learning classifiers using Gram-positive phyla data**


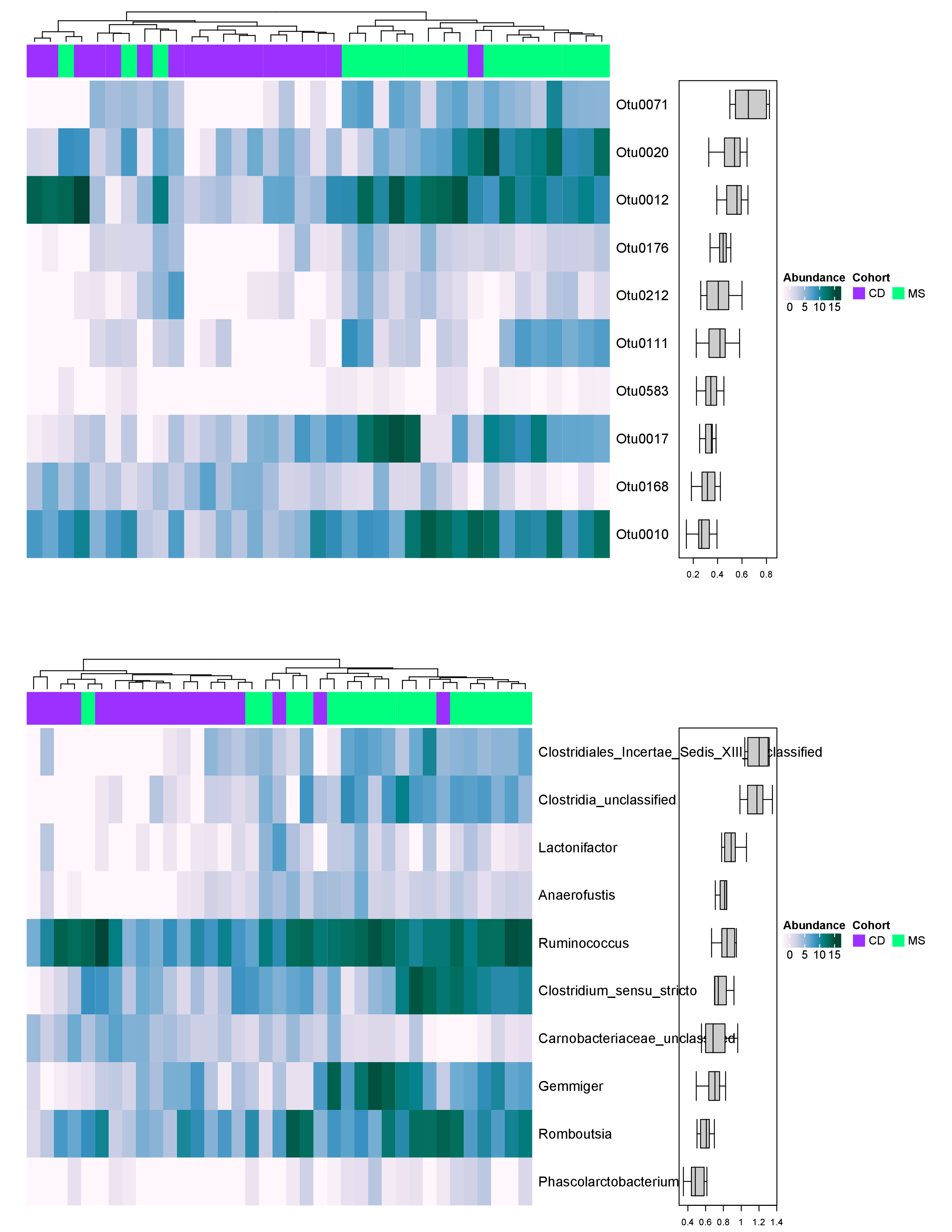

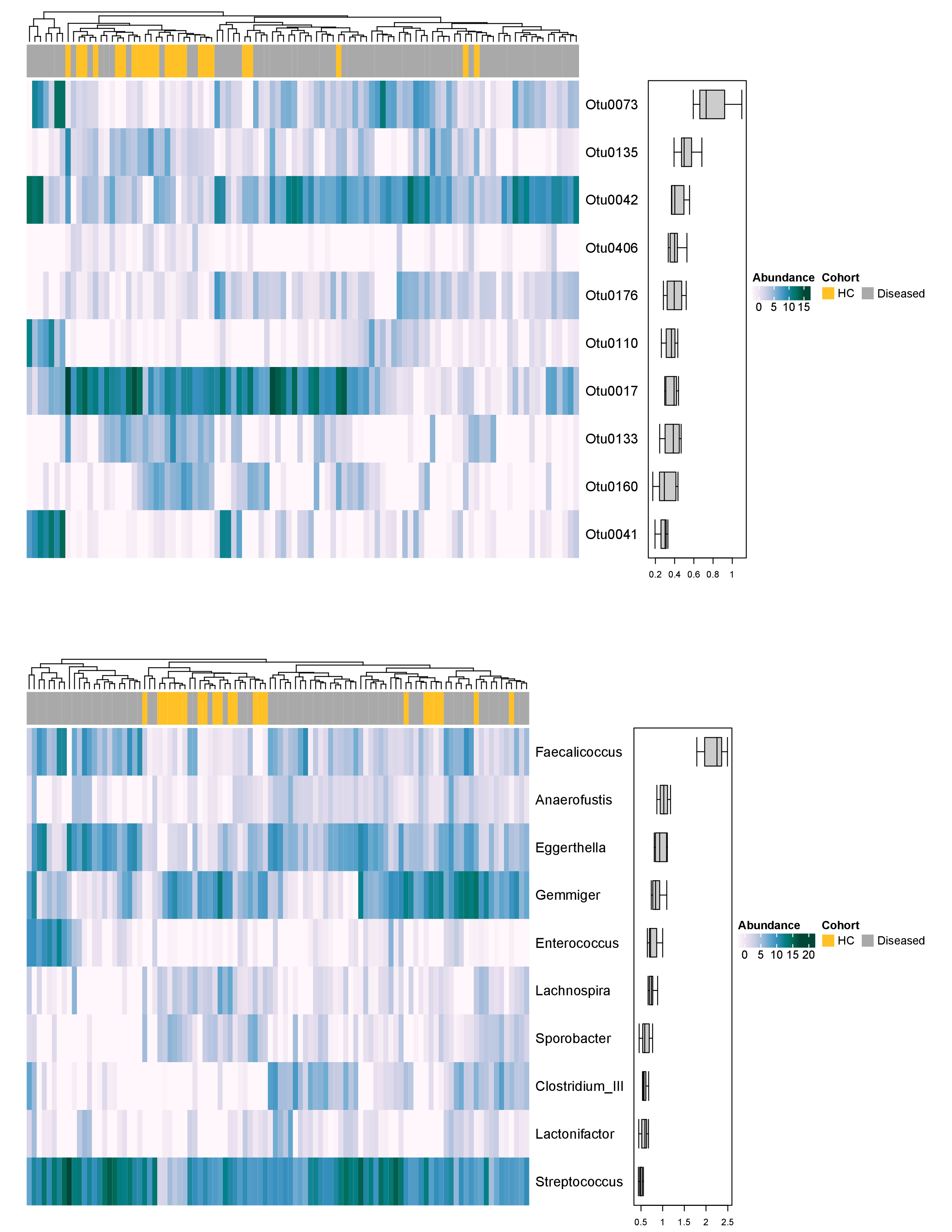

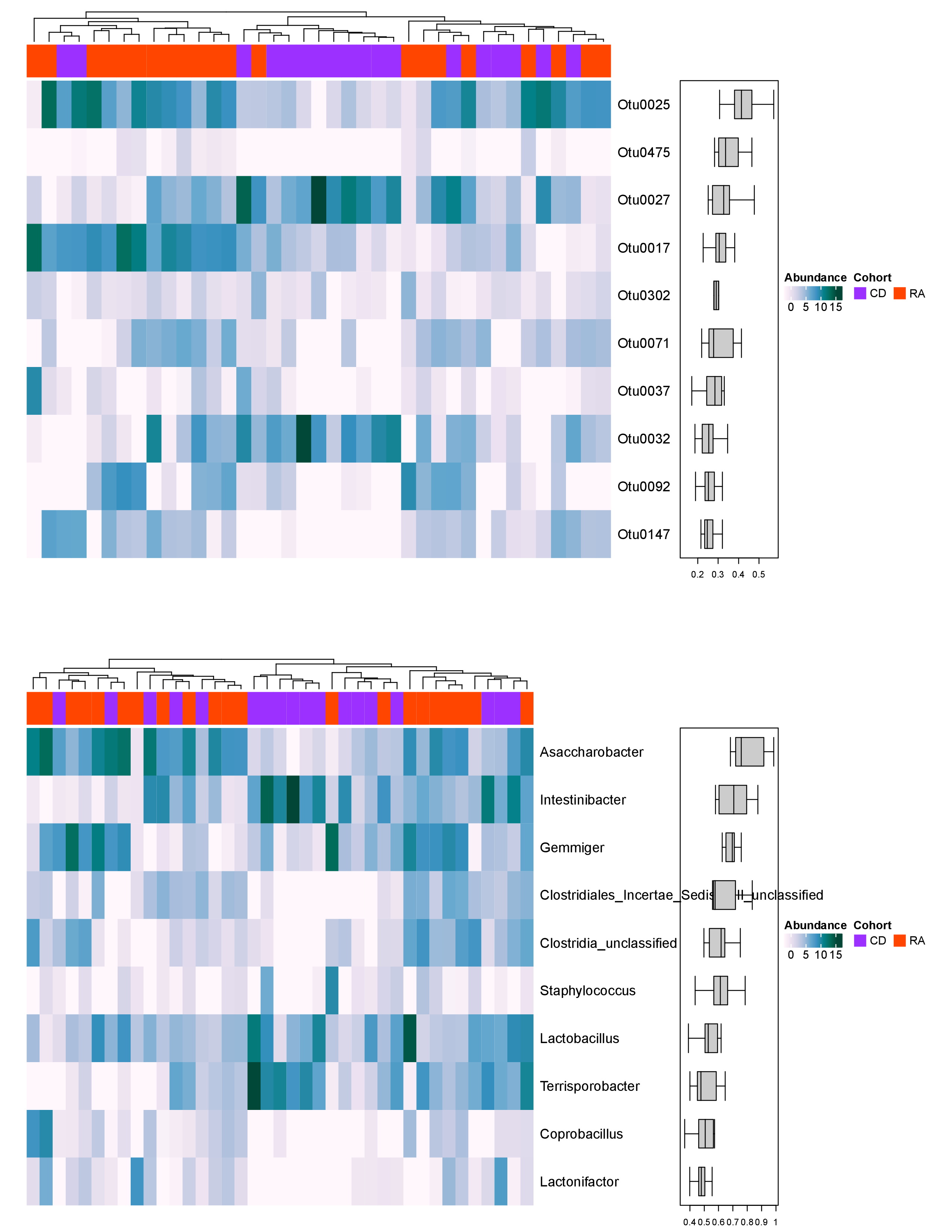

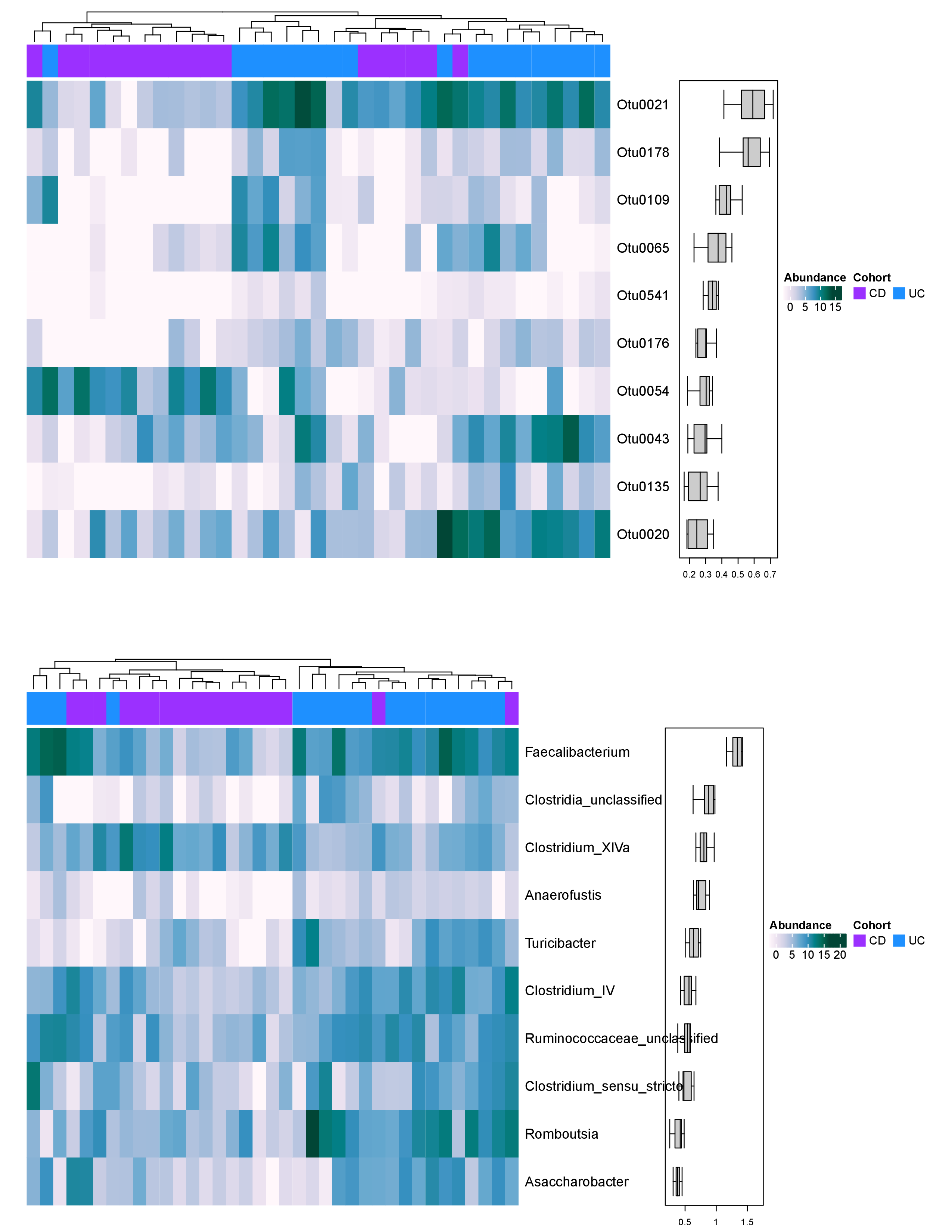

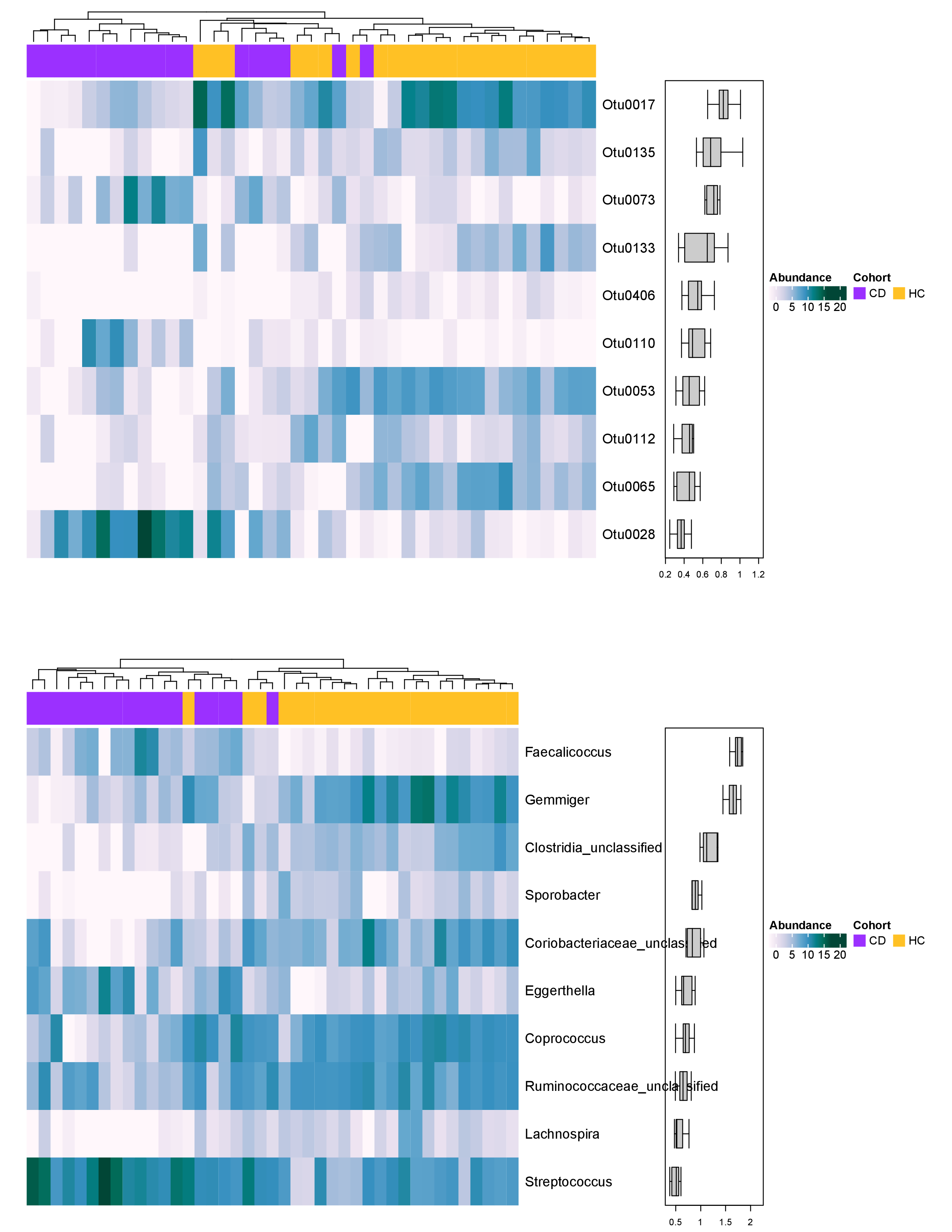

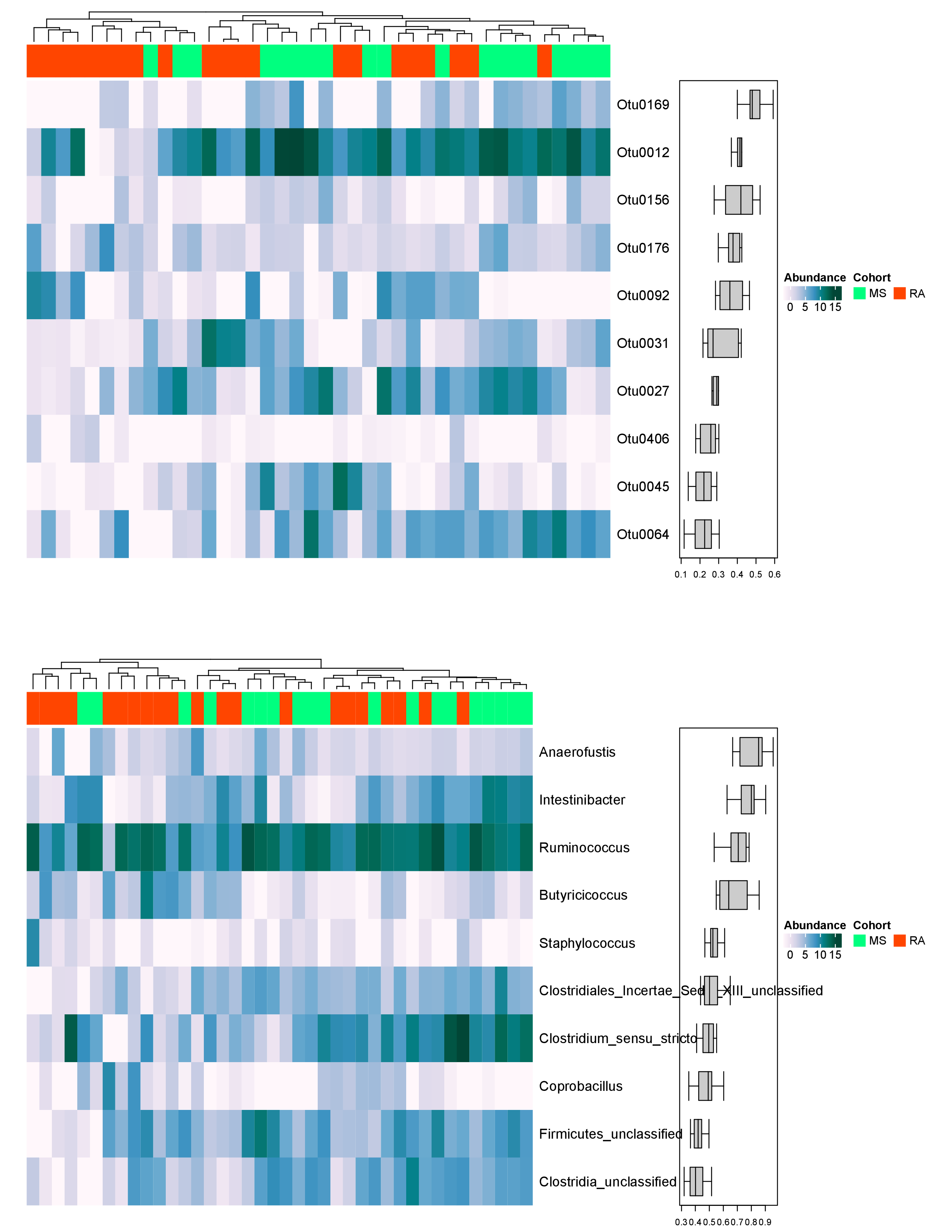

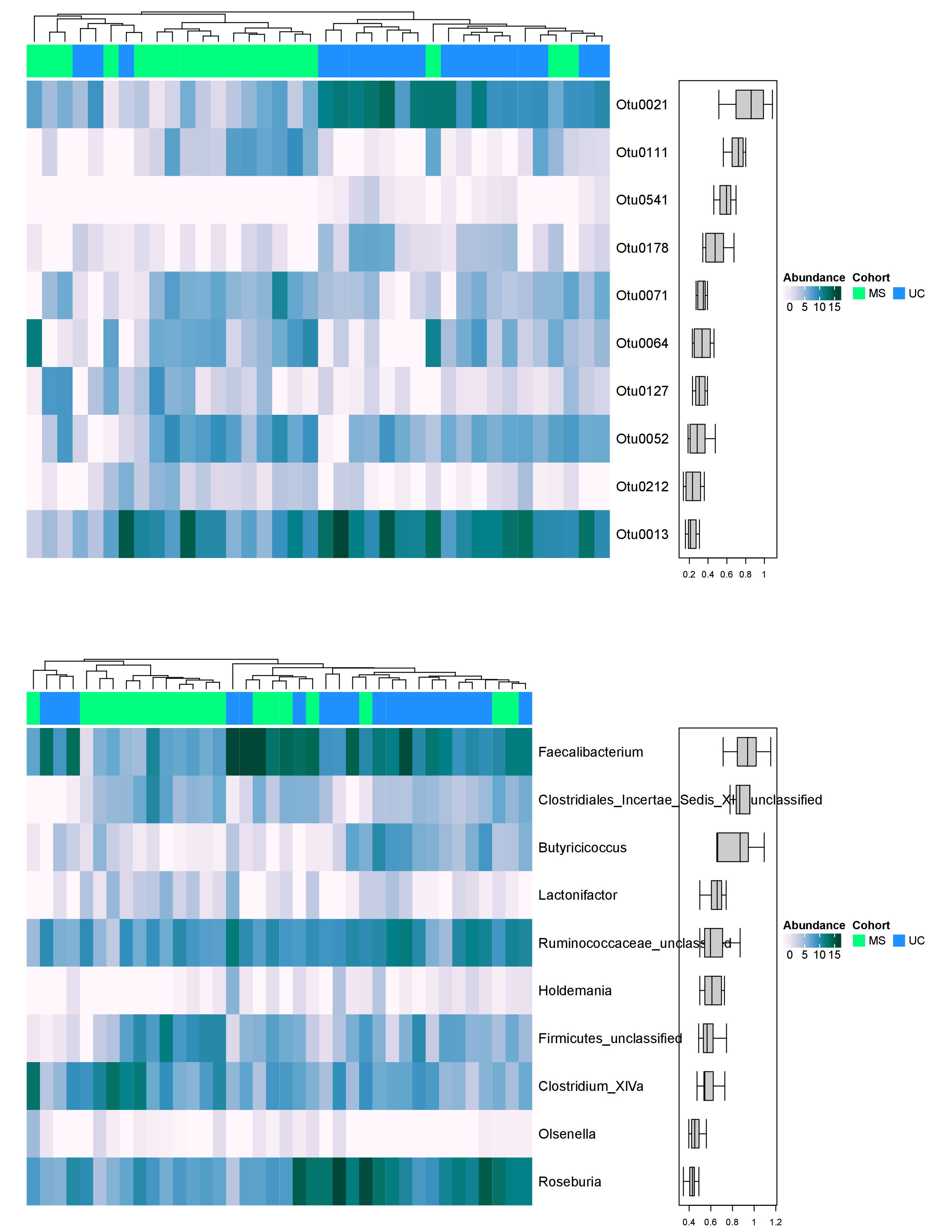

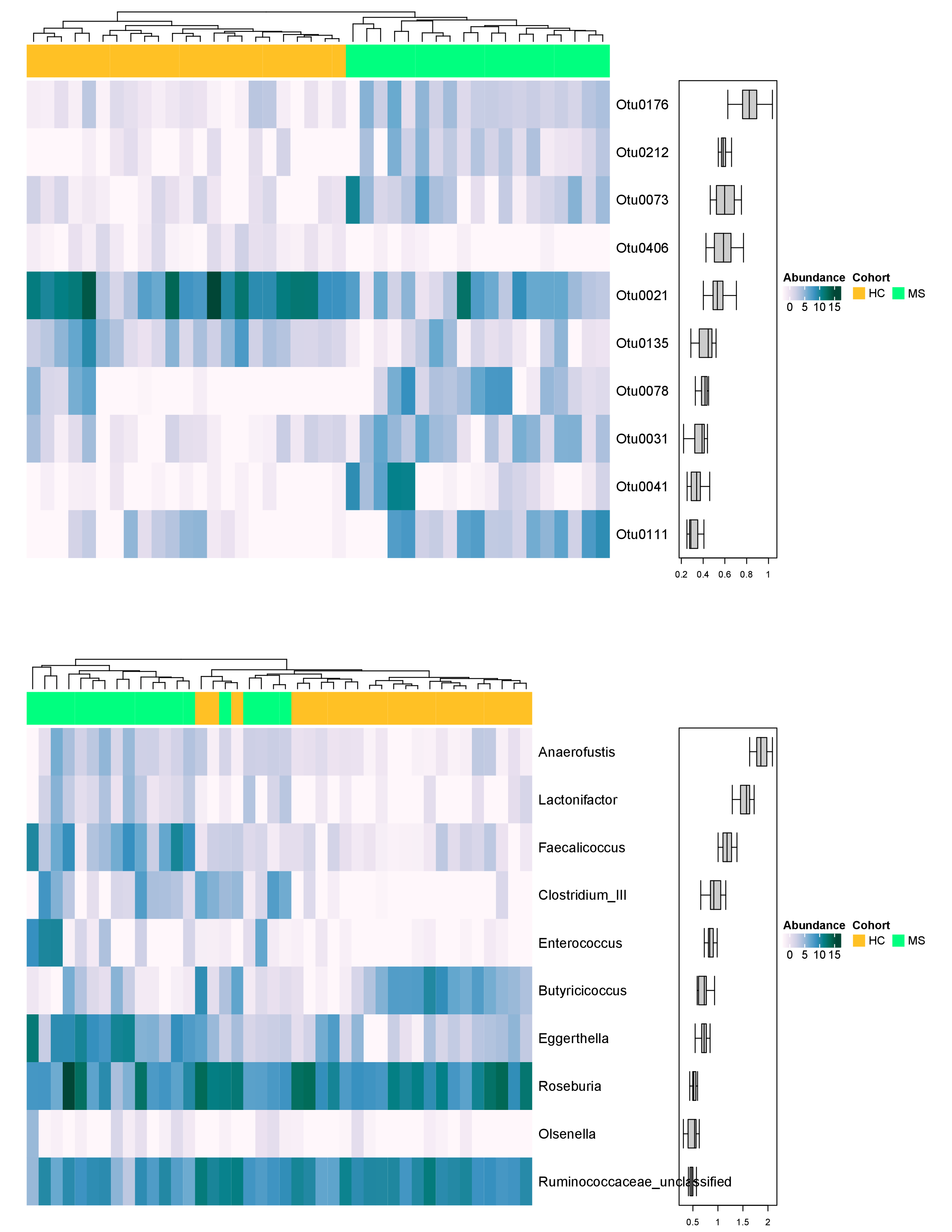

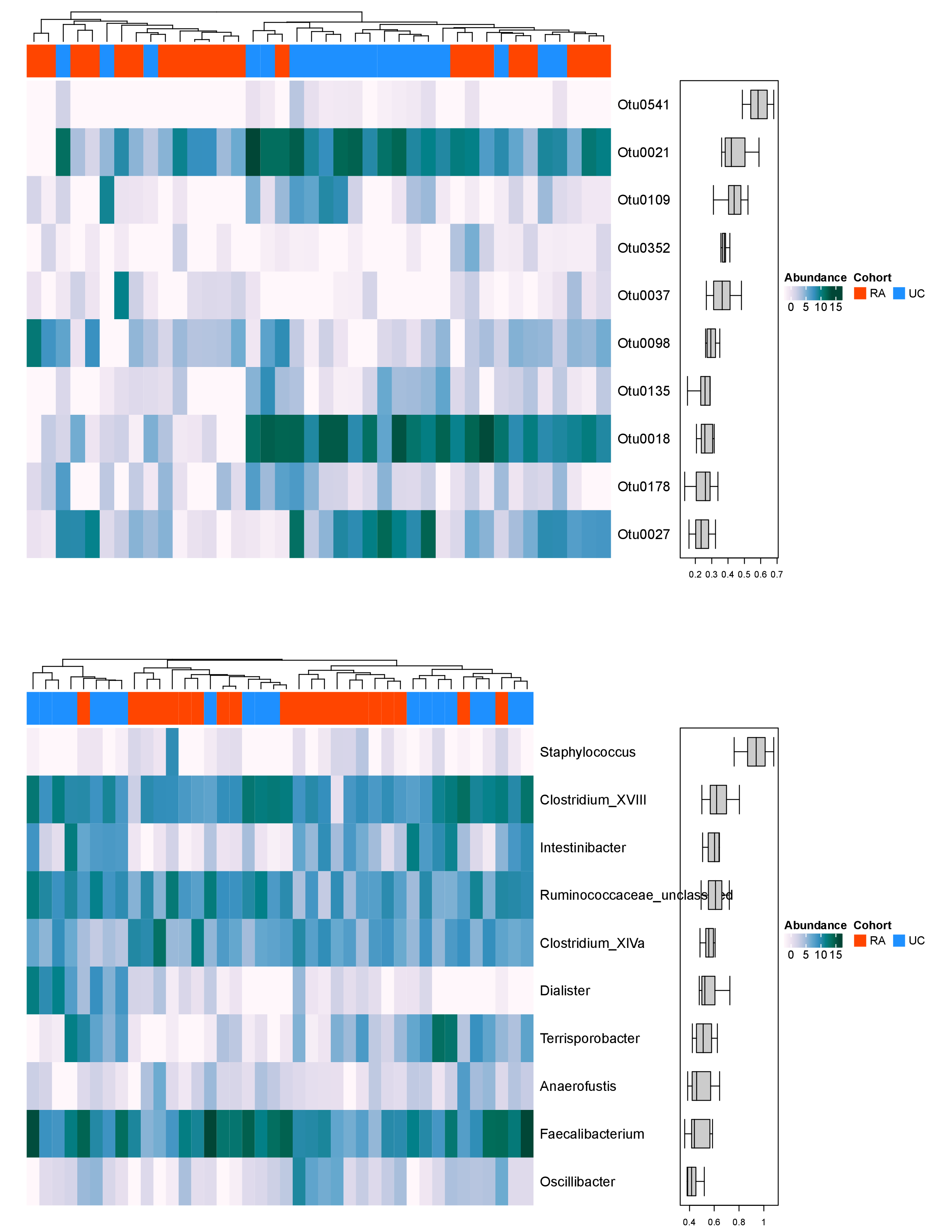

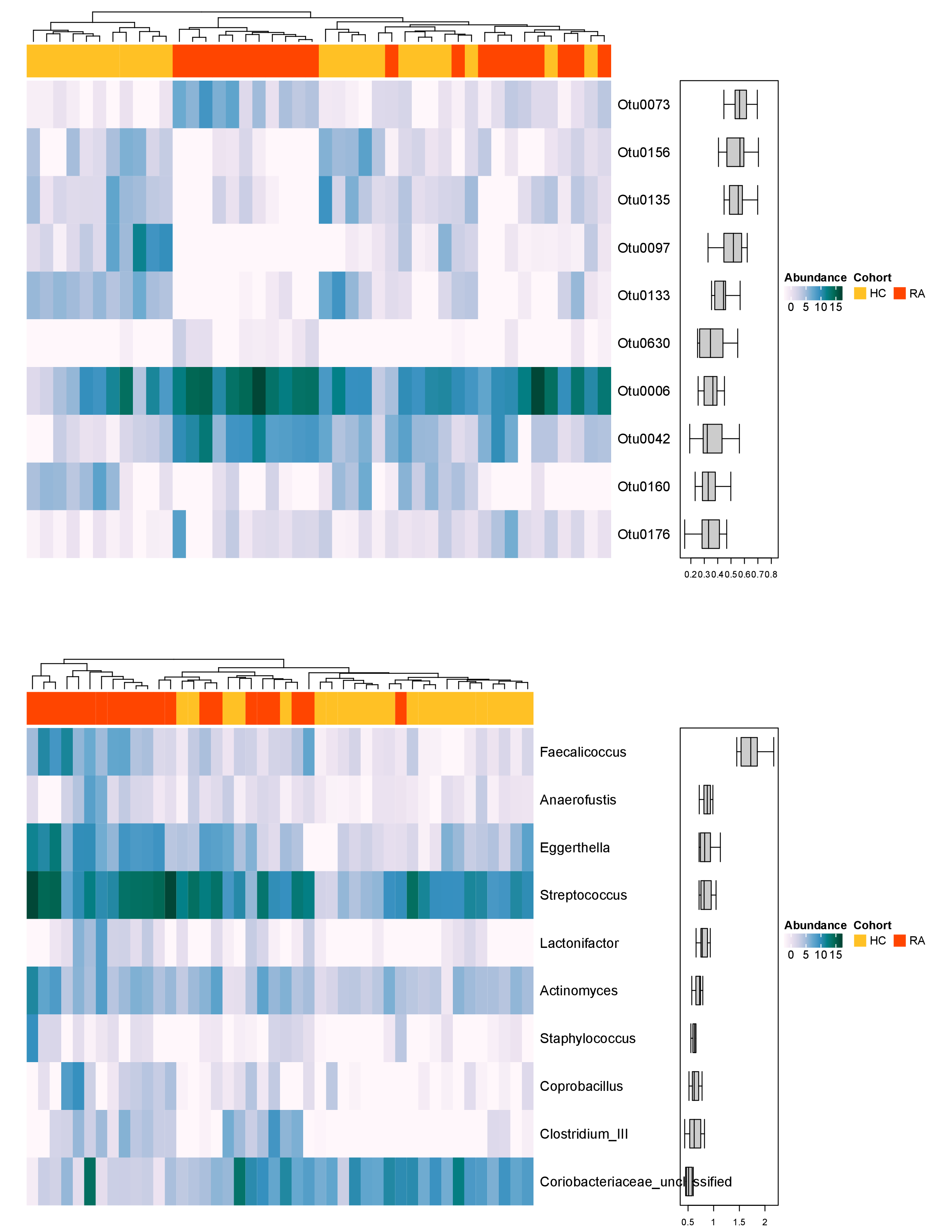

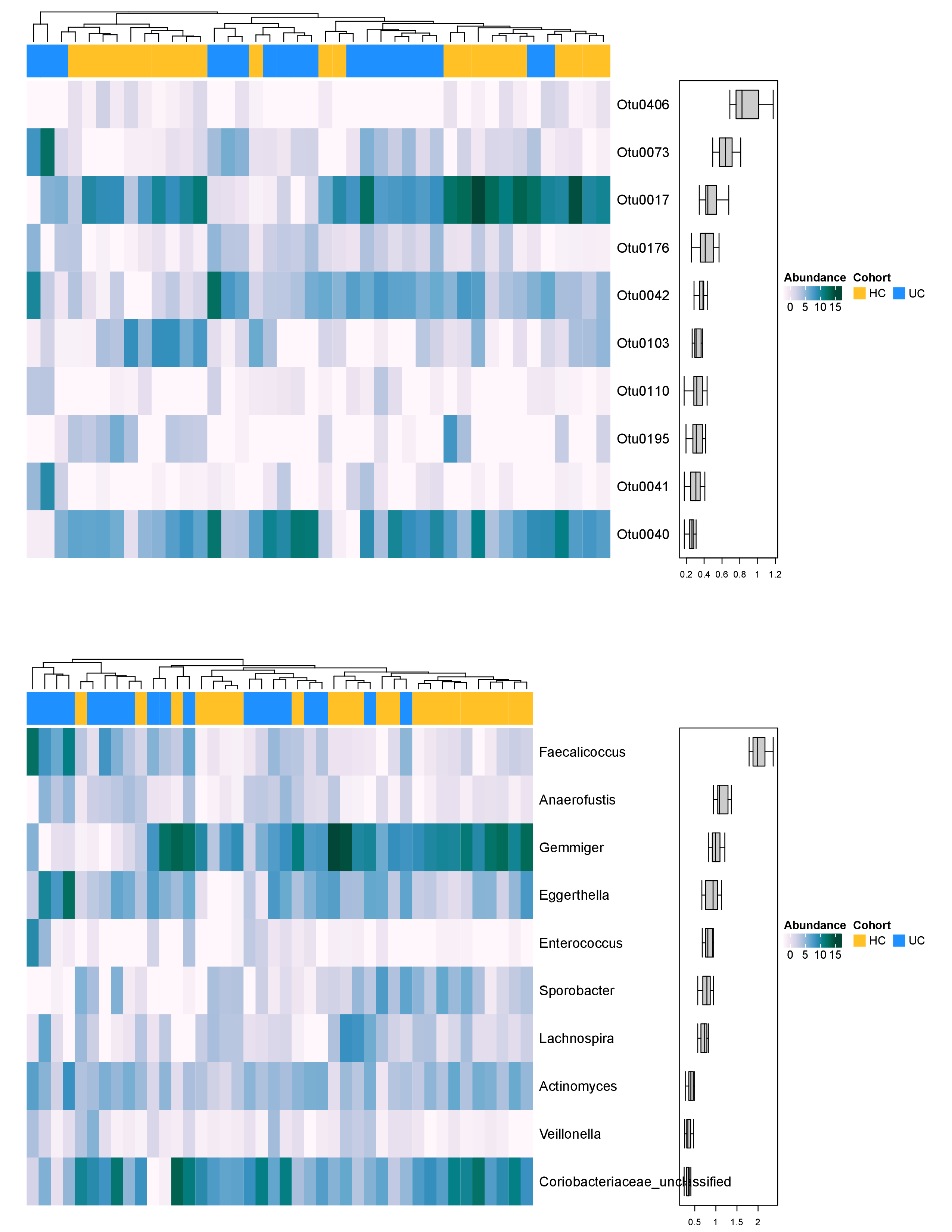

Supplement: Supplementary file 1 — Table S1. PERMANOVA analysis used to assess microbial community structure differences. Table S2. Median abundance of abundant taxa in IMID and HC microbiota. Table S3. Model performance of the binary classifiers trained on data using all phyla. Table S4. Model performance of the binary classifiers trained on data from the first biological replicate and predicted on the second biological replicate. Table S5. Importance of top 5 features in any of the OTU classifiers using Gram-positive phyla data. Table S6. Importance of top 5 features in any of the genus classifiers using Gram-positive phyla data. Figure S1. Sample distances shown in Bray-Curtis dissimilarity and principal coordinate analysis (PCoA) plot. Figure S2. Taxa identified as discriminating features for IMIDs versus HCs. Figure S3. Feature importance from pair-wise machine learning classifiers using all Phyla. Figure S4. Feature importance from pair-wise machine learning classifiers using Gram-positive phyla data. (DOCX 10707 kb) [file 40168_2018_603_MOESM1_ESM.docx]
